# Accuracy, robustness and scalability of dimensionality reduction methods for single-cell RNA-seq analysis

**DOI:** 10.1186/s13059-019-1898-6

**Published:** 2019-12-10

**Authors:** Shiquan Sun, Jiaqiang Zhu, Ying Ma, Xiang Zhou

**Affiliations:** 1 School of Computer Science, Northwestern Polytechnical University, Xi’an, Shaanxi 710072 People’s Republic of China; 2 Department of Biostatistics, University of Michigan, Ann Arbor, MI 48109 USA; 3 Center for Statistical Genetics, University of Michigan, Ann Arbor, MI 48109 USA

## Abstract

**Background:**

Dimensionality reduction is an indispensable analytic component for many areas of single-cell RNA sequencing (scRNA-seq) data analysis. Proper dimensionality reduction can allow for effective noise removal and facilitate many downstream analyses that include cell clustering and lineage reconstruction. Unfortunately, despite the critical importance of dimensionality reduction in scRNA-seq analysis and the vast number of dimensionality reduction methods developed for scRNA-seq studies, few comprehensive comparison studies have been performed to evaluate the effectiveness of different dimensionality reduction methods in scRNA-seq.

**Results:**

We aim to fill this critical knowledge gap by providing a comparative evaluation of a variety of commonly used dimensionality reduction methods for scRNA-seq studies. Specifically, we compare 18 different dimensionality reduction methods on 30 publicly available scRNA-seq datasets that cover a range of sequencing techniques and sample sizes. We evaluate the performance of different dimensionality reduction methods for neighborhood preserving in terms of their ability to recover features of the original expression matrix, and for cell clustering and lineage reconstruction in terms of their accuracy and robustness. We also evaluate the computational scalability of different dimensionality reduction methods by recording their computational cost.

**Conclusions:**

Based on the comprehensive evaluation results, we provide important guidelines for choosing dimensionality reduction methods for scRNA-seq data analysis. We also provide all analysis scripts used in the present study at www.xzlab.org/reproduce.html.

## Introduction

Single-cell RNA sequencing (scRNA-seq) is a rapidly growing and widely applying technology [1–3]. By measuring gene expression at a single-cell level, scRNA-seq provides an unprecedented opportunity to investigate the cellular heterogeneity of complex tissues [4–8]. However, despite the popularity of scRNA-seq, analyzing scRNA-seq data remains a challenging task. Specifically, due to the low capture efficiency and low sequencing depth per cell in scRNA-seq data, gene expression measurements obtained from scRNA-seq are noisy: collected scRNA-seq gene measurements are often in the form of low expression counts, and in studies not based on unique molecular identifiers, are also paired with an excessive number of zeros known as dropouts [9]. Subsequently, dimensionality reduction methods that transform the original high-dimensional noisy expression matrix into a low-dimensional subspace with enriched signals become an important data processing step for scRNA-seq analysis [10]. Proper dimensionality reduction can allow for effective noise removal, facilitate data visualization, and enable efficient and effective downstream analysis of scRNA-seq [11].

Dimensionality reduction is indispensable for many types of scRNA-seq analysis. Because of the importance of dimensionality reduction in scRNA-seq analysis, many dimensionality reduction methods have been developed and are routinely used in scRNA-seq software tools that include, but not limited to, cell clustering tools [12, 13] and lineage reconstruction tools [14]. Indeed, most commonly used scRNA-seq clustering methods rely on dimensionality reduction as the first analytic step [15]. For example, Seurat applies clustering algorithms directly on a low-dimensional space inferred from principal component analysis (PCA) [16]. CIDR improves clustering by improving PCA through imputation [17]. SC3 combines different ways of PCA for consensus clustering [18]. Besides PCA, other dimensionality reduction techniques are also commonly used for cell clustering. For example, nonnegative matrix factorization (NMF) is used in SOUP [19]. Partial least squares is used in scPLS [20]. Diffusion map is used in destiny [21]. Multidimensional scaling (MDS) is used in ascend [22]. Variational inference autoencoder is used in scVI [23]. In addition to cell clustering, most cell lineage reconstruction and developmental trajectory inference algorithms also rely on dimensionality reduction [14]. For example, TSCAN builds cell lineages using minimum spanning tree based on a low-dimensional PCA space [24]. Waterfall performs *k*-means clustering in the PCA space to eventually produce linear trajectories [25]. SLICER uses locally linear embedding (LLE) to project the set of cells into a lower-dimension space for reconstructing complex cellular trajectories [26]. Monocle employs either independent components analysis (ICA) or uniform manifold approximation and projection (UMAP) for dimensionality reduction before building the trajectory [27, 28]. Wishbone combines PCA and diffusion maps to allow for bifurcation trajectories [29].

Besides the generic dimensionality reduction methods mentioned in the above paragraph, many dimensionality reduction methods have also been developed recently that are specifically targeted for modeling scRNA-seq data. These scRNA-seq-specific dimensionality reduction methods can account for either the count nature of scRNA-seq data and/or the dropout events commonly encountered in scRNA-seq studies. For example, ZIFA relies on a zero-inflation normal model to model dropout events [30]. pCMF models both dropout events and the mean-variance dependence resulting from the count nature of scRNA-seq data [31]. ZINB-WaVE incorporates additional gene-level and sample-level covariates for more accurate dimensionality reduction [32]. Finally, several deep learning-based dimensionality reduction methods have recently been developed to enable scalable and effective computation in large-scale scRNA-seq data, including data that are collected by 10X Genomics techniques [33] and/or from large consortium studies such as Human Cell Atlas (HCA) [34, 35]. Common deep learning-based dimensionality reduction methods for scRNA-seq include Dhaka [36], scScope [37], VASC [38], scvis [39], and DCA [40], to name a few.

With all these different dimensionality reduction methods for scRNA-seq data analysis, one naturally wonders which dimensionality reduction method one would prefer for different types of scRNA-seq analysis. Unfortunately, despite the popularity of scRNA-seq technique, the critical importance of dimensionality reduction in scRNA-seq analysis, and the vast number of dimensionality reduction methods developed for scRNA-seq studies, few comprehensive comparison studies have been performed to evaluate the effectiveness of different dimensionality reduction methods for practical applications. Here, we aim to fill this critical knowledge gap by providing a comprehensive comparative evaluation of a variety of commonly used dimensionality reduction methods for scRNA-seq studies. Specifically, we compared 18 different dimensionality reduction methods on 30 publicly available scRNA-seq data sets that cover a range of sequencing techniques and sample sizes [12, 14, 41]. We evaluated the performance of different dimensionality reduction methods for neighborhood preserving in terms of their ability to recover features of the original expression matrix, and for cell clustering and lineage reconstruction in terms of their accuracy and robustness using different metrics. We also evaluated the computational scalability of different dimensionality reduction methods by recording their computational time. Together, we hope our results can serve as an important guideline for practitioners to choose dimensionality reduction methods in the field of scRNA-seq analysis.

## Results

**Fig. 1:**
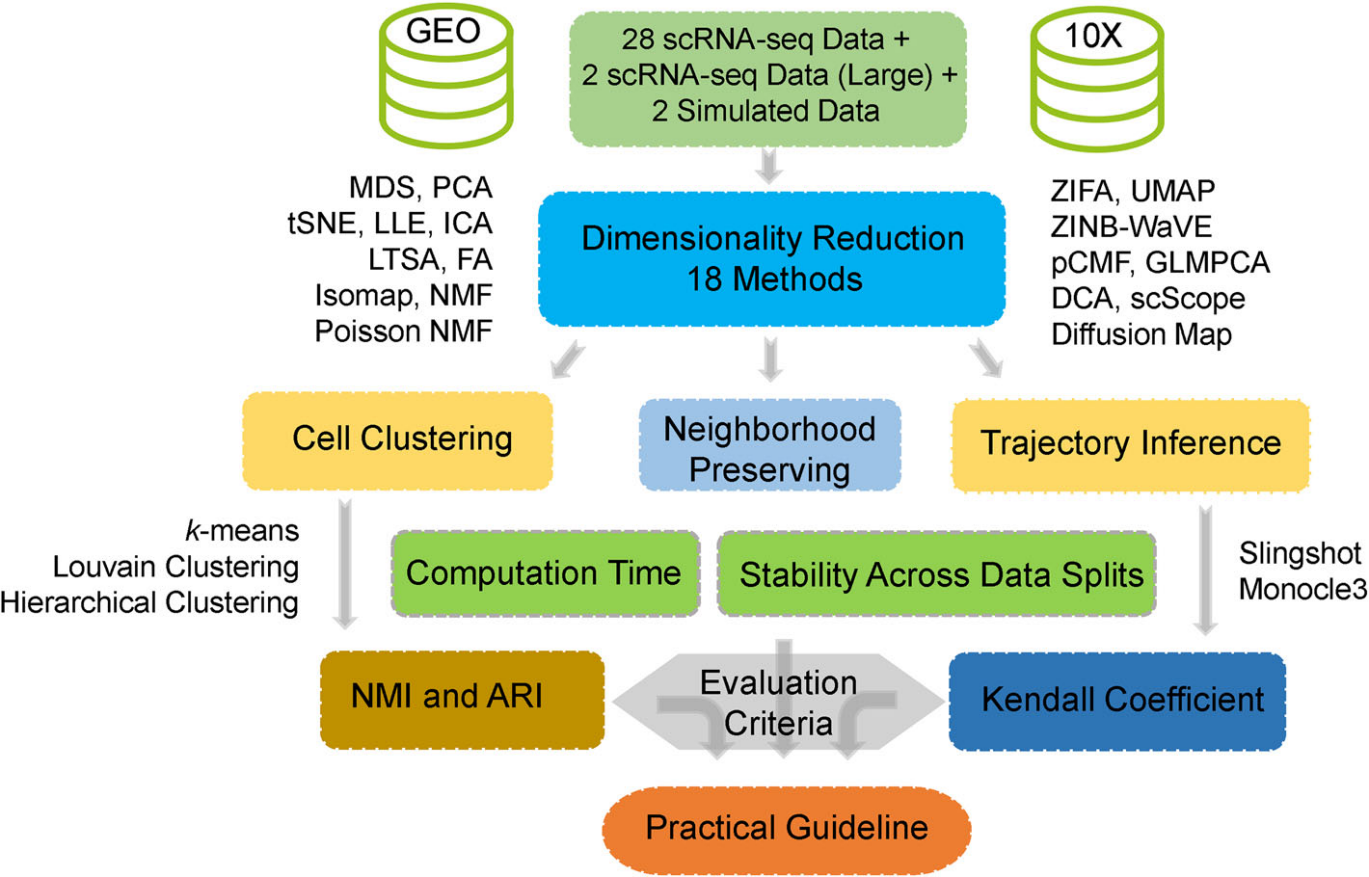
Overview of the evaluation workflow for dimensionality reduction methods. We obtained a total of 30 publicly available scRNA-seq data from GEO and 10X Genomics website. We also simulated two addition simulation data sets. For each of the 32 data sets in turn, we applied 18 dimensionality reduction methods to extract the low-dimensional components. Afterwards, we evaluated the performance of dimensionality reduction methods by evaluating how effective the low-dimensional components extracted from dimensionality reduction methods are for downstream analysis. We did so by evaluating the two commonly applied downstream analysis: clustering analysis and lineage reconstruction analysis. In the analysis, we varied the number of low-dimensional components extracted from these dimensionality reduction methods. The performance of each dimensionality reduction method is qualified by Jaccard index for neighborhood preserving, normalized mutual information (NMI) and adjusted rand index (ARI) for cell clustering analysis, and Kendall correlation coefficient for trajectory inference. We also recorded the stability of each dimensionality reduction method across data splits and recorded the computation time for each dimensionality reduction method. Through the comprehensive evaluation, we eventually provide practical guidelines for practitioners to choose dimensionality reduction methods for scRNA-seq data analysis

We evaluated the performance of 18 dimensionality reduction methods (Table 1; Additional file 1: Figure S1) on 30 publicly available scRNA-seq data sets (Additional file 1: Table S1-S2) and 2 simulated data sets. Details of these data sets are provided in “Methods and Materials.” Briefly, these data sets cover a wide variety of sequencing techniques that include Smart-Seq2 [1] (8 data sets), Smart-Seq [53] (5 data sets), 10X Genomics [33] (6 data sets), inDrop [54] (1 data set), RamDA-seq [55] (1 data set), sci-RNA-seq3 [28] (1 data set), SMARTer [56] (5 data sets), and others [57] (3 data sets). In addition, these data sets cover a range of sample sizes from a couple of hundred cells to over tens of thousands of cells. In each data set, we evaluated the ability of different dimensionality reduction methods in preserving the original feature of the expression matrix, and, more importantly, their effectiveness for two important single-cell analytic tasks: cell clustering and lineage inference. In particular, we used 14 real data sets together with 2 simulated data sets for dimensionality reduction method comparison in terms of cell clustering performance. We used another set of 14 real data sets for dimensionality reduction method comparison in terms of trajectory inference. We used yet two additional large-scale scRNA-seq data sets to examine the effectiveness and scalability of different dimensionality reduction methods there. In addition, we measured the computing stability of different dimensionality reduction methods and recorded their computation time. An overview of the comparison workflow is shown in Fig. 1. Because common tSNE software can only extract a small number low-dimensional components [48, 58, 59], we only included tSNE results based on two low-dimensional components extracted from the recently developed fast *FIt-SNE* R package [48] in all figures. All data and analysis scripts for reproducing the results in the paper are available at www.xzlab.org/reproduce.html or https://github.com/xzhoulab/DRComparison.

**Table 1:** List of compared dimensionality reduction methods. We list standard modeling properties for each of compared dimensionality reduction methods

No.	Methods	Modeling counts	Modeling zero inflation	Non-linear projection	Computation efficiency	Implementation language	Year of publication	Reference
1	PCA	No	No	No	Yes	R	1901	[42]
2	ICA	No	No	No	No	R	1994	[43]
3	FA	No	No	No	Yes	R	1952	[44]
4	NMF	No	No	No	Yes	R	1999	[45]
5	Poisson NMF	Yes	No	No	Yes	R	1999	[45]
6	Diffusion Map	No	No	Yes	Yes	R	2005	[46]
7	ZIFA	No	Yes	No	No	Python	2016	[30]
8	ZINB-WaVE	Yes	Yes	No	No	R	2018	[32]
9	GLMPCA	Yes	No	No	No	R	2019	[47]
10	pCMF	Yes	Yes	No	No	R	2019	[31]
11	scScope	No	Yes	Yes	Yes	Python	2019	[37]
12	DCA	Yes	Yes	Yes	Yes	Python	2018	[40]
13	tSNE	No	No	Yes	No	R	2008	[48]
14	MDS	No	No	No	Yes	R	1958	[49]
15	LLE	No	No	Yes	Yes	R	2000	[50]
16	LTSA	No	No	Yes	No	R	2004	[51]
17	Isomap	No	No	Yes	Yes	R	2000	[11]
18	UMAP	No	No	Yes	Yes	Python	2019	[52]

These properties include whether it models count data (3rd column), whether it accounts for zero inflation (4th column), whether it is a linear dimensionality reduction method (5th column), its computation efficiency (6th column), implementation language (7th column), year of publication (8th column), and reference (9th column). *FA* factor analysis, *PCA* principal component analysis, *ICA* independent component analysis, *NMF* nonnegative matrix factorization, *Poisson NMF* Kullback-Leibler divergence-based NMF, *ZIFA* zero-inflated factor analysis, *ZINB-WaVE* zero-inflated negative binomial-based wanted variation extraction, *pCMF* probabilistic count matrix factorization, *DCA* deep count autoencoder network, *scScope* scalable deep-learning-based approach, *GLMPCA* generalized linear model principal component analysis, Diffusion Map, *MDS* multidimensional scaling, *LLE* locally linear embedding, *LTSA* local tangent space alignment, Isomap; *UMAP* uniform manifold approximation and projection, *tSNE t*-distributed stochastic neighbor embedding

### Performance of dimensionality reduction methods for neighborhood preserving

We first evaluated the performance of different dimensionality reduction methods in terms of preserving the original features of the gene expression matrix. To do so, we applied different dimensionality reduction methods to each of 30 scRNA-seq data sets (28 real data and 2 simulated data; excluding the two large-scale data due to computing concerns) and evaluated the performance of these dimensionality reduction methods based on neighborhood preserving. Neighborhood preserving measures how the local neighborhood structure in the reduced dimensional space resembles that in the original space by computing a Jaccard index [60] (details in “Methods and Materials”). In the analysis, for each dimensionality reduction method and each scRNA-seq data set, we applied the dimensionality reduction method to extract a fixed number of low-dimensional components (e.g., these are the principal components in the case of PCA). We varied the number of low-dimensional components to examine their influence on local neighborhood preserving. Specifically, for each of 16 cell clustering data sets, we varied the number of low-dimensional components to be either 2, 6, 14, or 20 when the data contains less than or equal to 300 cells, and we varied the number of low-dimensional components to be either 0.5%, 1%, 2%, or 3% of the total number of cells when the data contains more than 300 cells. For each of the 14 trajectory inference data sets, we varied the number of low-dimensional components to be either 2, 6, 14, or 20 regardless of the number of cells. Finally, we also varied the number of neighborhood cells used in the Jaccard index to be either 10, 20, or 30. The evaluation results based on the Jaccard index of neighborhood preserving are summarized in Additional file 1: Figure S2-S14.

In the cell clustering data sets, we found that pCMF achieves the best performance of neighborhood preserving across all data sets and across all included low-dimensional components (Additional file 1: Figure S2-S7). For example, with 30 neighborhood cells and 0.5% of low-dimensional components, pCMF achieves a Jaccard index of 0.25. Its performance is followed by Poisson NMF (0.16), ZINB-WaVE (0.16), Diffusion Map (0.16), MDS (0.15), and tSNE (0.14). While the remaining two methods, scScope (0.1) and LTSA (0.06), do not fare well. Increasing number of neighborhood cells increases the absolute value of Jaccard index but does not influence the relative performance of dimensionality reduction methods (Additional file 1: Figure S7). In addition, the relative performance of most dimensionality reduction methods remains largely similarly whether we focus on data sets with unique molecular identifiers (UMI) or data sets without UMI (Additional file 1: Figure S8). However, we do notice two exceptions: the performance of pCMF decreases with increasing number of low-dimensional components in UMI data but increases in non-UMI data; the performance of scScope is higher in UMI data than its performance in non-UMI data. In the trajectory inference data sets, pCMF again achieves the best performance of neighborhood preserving across all data sets and across all included low-dimensional components (Additional file 1: Figure S9-S14). Its performance is followed closely by scScope and Poisson NMF. For example, with 30 neighborhood cells and 20 low-dimensional components, the Jaccard index of pCMF, Poisson NMF, and scScope across all data sets are 0.3, 0.28, and 0.26, respectively. Their performance is followed by ZINB-WaVE (0.19), FA (0.18), ZIFA (0.18), GLMPCA (0.18), and MDS (0.18). In contrast, LTSA also does not fare well across all included low-dimensional components (Additional file 1: Figure S14). Again, increasing number of neighborhood cells increases the absolute value of Jaccard index but does not influence the relative performance among dimensionality reduction methods (Additional file 1: Figure S9-S14).

We note that the measurement we used in this subsection, neighborhood preserving, is purely for measuring dimensionality reduction performance in terms of preserving the original gene expression matrix and may not be relevant for single-cell analytic tasks that are the main focus of the present study: a dimensionality reduction method that preserves the original gene expression matrix may not be effective in extracting useful biological information from the expression matrix that is essential for key downstream single-cell applications. Preserving the original gene expression matrix is rarely the sole purpose of dimensionality reduction methods for single-cell applications: indeed, the original gene expression matrix (which is the best-preserved matrix of itself) is rarely, if ever, used directly in any downstream single-cell applications including clustering and lineage inference, even though it is computationally easy to do so. Therefore, we will focus our main comparison in two important downstream single-cell applications listed below.

### Performance of dimensionality reduction methods for cell clustering

As our main comparison, we first evaluated the performance of different dimensionality reduction methods for cell clustering applications. To do so, we obtained 14 publicly available scRNA-seq data sets and simulated two additional scRNA-seq data sets using the *Splatter* package (Additional file 1: Table S1). Each of the 14 real scRNA-seq data sets contains known cell clustering information while each of the 2 simulated data sets contains 4 or 8 known cell types. For each dimensionality reduction method and each data set, we applied dimensionality reduction to extract a fixed number of low-dimensional components (e.g., these are the principal components in the case of PCA). We again varied the number of low-dimensional components as in the previous section to examine their influence on cell clustering analysis. We then applied either the hierarchical clustering method, the *k*-means clustering method, or Louvain clustering method [61] to obtain the inferred cluster labels. We used both normalized mutual information (NMI) and adjusted rand index (ARI) values for comparing the true cell labels and inferred cell labels obtained by clustering methods based on the low-dimensional components.

#### Cell clustering with different clustering methods

**Fig. 2:**
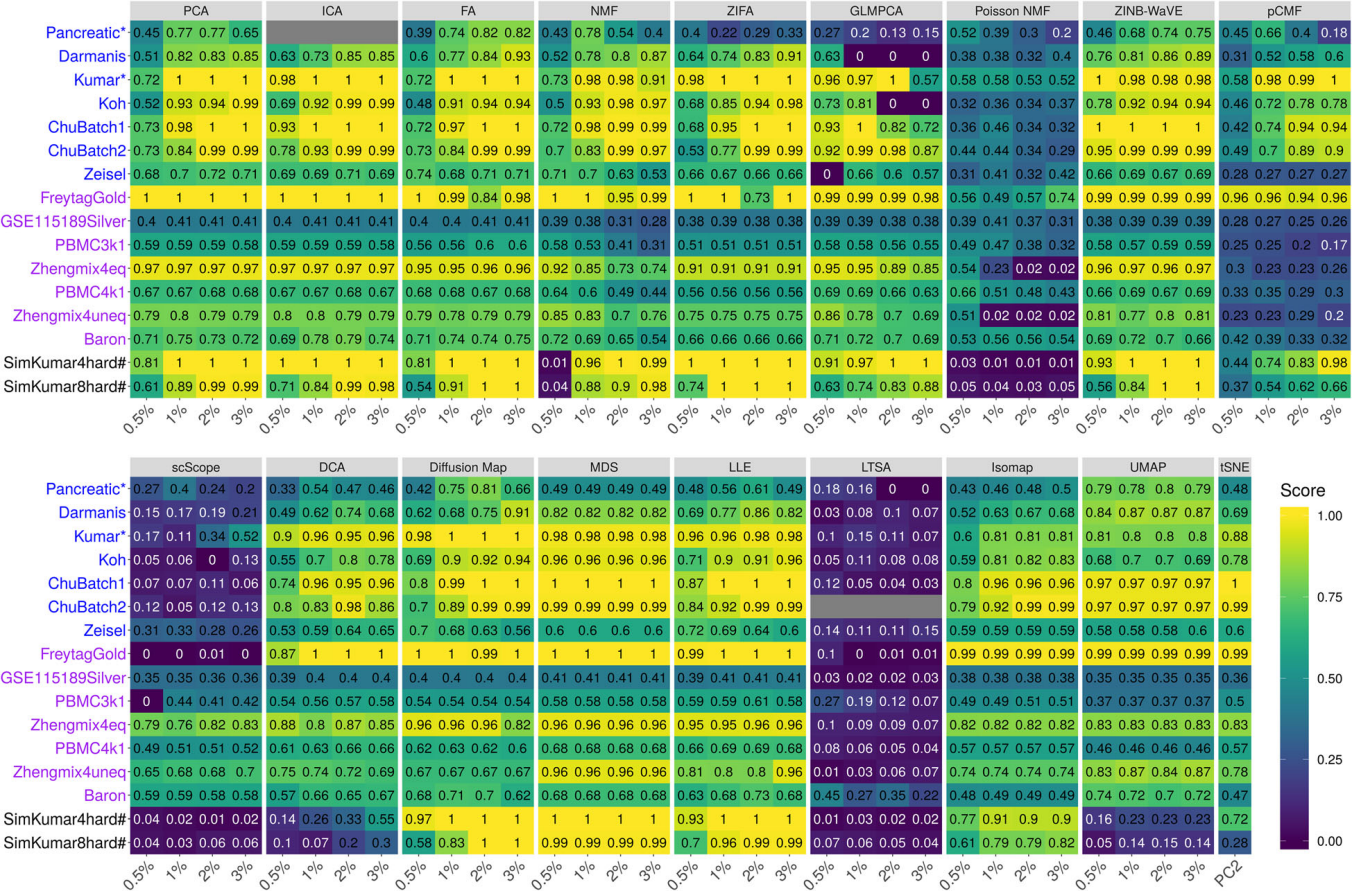
Dimensionality reduction method performance evaluated by *k*-means clustering based on NMI in downstream cell clustering analysis. We compared 18 dimensionality reduction methods (columns), including factor analysis (FA), principal component analysis (PCA), independent component analysis (ICA), Diffusion Map, nonnegative matrix factorization (NMF), Poisson NMF, zero-inflated factor analysis (ZIFA), zero-inflated negative binomial based wanted variation extraction (ZINB-WaVE), probabilistic count matrix factorization (pCMF), deep count autoencoder network (DCA), scScope, generalized linear model principal component analysis (GLMPCA), multidimensional scaling (MDS), locally linear embedding (LLE), local tangent space alignment (LTSA), Isomap, uniform manifold approximation and projection (UMAP), and *t*-distributed stochastic neighbor embedding (tSNE). We evaluated their performance on 14 real scRNA-seq data sets (UMI-based data are labeled as purple; non-UMI-based data are labeled as blue) and 2 simulated data sets (rows). The simulated data based on Kumar data is labeled with #. The performance of each dimensionality reduction method is measured by normalized mutual information (NMI). For each data set, we compared the four different numbers of low-dimensional components. The four numbers equal to 0.5%, 1%, 2%, and 3% of the total number of cells in big data and equal to 2, 6, 14, and 20 in small data (which are labeled with*). For convenience, we only listed 0.5%, 1%, 2%, and 3% on *x*-axis. No results for ICA are shown in the table (gray fills) because ICA cannot handle the large number of features in that data. No results for LTSA are shown (gray fills) because error occurred when we applied the clustering method on LTSA extracted low-dimensional components there. Note that, for tSNE, we only extracted two low-dimensional components due to the limitation of the tSNE software

The evaluation results on dimensionality reduction methods based on clustering analysis using the *k*-means clustering algorithm are summarized in Fig. 2 (for NMI criterion) and Additional file 1: Figure S15 (for ARI criterion). Because the results based on either of the two criteria are similar, we will mainly explain the results based on the NMI criteria in Fig. 2. For easy visualization, we also display the results averaged across data sets in Additional file 1: Figure S16. A few patterns are noticeable. First, as one would expect, clustering accuracy depends on the number of low-dimensional components that are used for clustering. Specifically, accuracy is relatively low when the number of included low-dimensional components is very small (e.g., 2 or 0.5%) and generally increases with the number of included components. In addition, accuracy usually saturates once a sufficient number of components is included, though the saturation number of components can vary across data sets and across methods. For example, the average NMI across all data sets and across all methods are 0.61, 0.66, 0.67, and 0.67 for increasingly large number of components, respectively. Second, when conditional on using a low number of components, scRNA-seq-specific dimensionality reduction method ZINB-WaVE and generic dimensionality reduction methods ICA and MDS often outperform the other methods. For example, with the lowest number of components, the average NMI across all data sets for MDS, ICA, and ZINB-WaVE are 0.82, 0.77 and 0.76, respectively (Additional file 1: Figure S16A). The performance of MDS, ICA, and ZINB-WaVE is followed by LLE (0.75), Diffusion Map (0.71), ZIFA (0.69), PCA (0.68), FA (0.68), tSNE (0.68), NMF (0.59), and DCA (0.57). While the remaining four methods, Poisson NMF (0.42), pCMF (0.41), scScope (0.26), and LTSA (0.12), do not fare well with a low number of components. Third, with increasing number of low-dimensional components, generic methods such as FA, ICA, MDS, and PCA are often comparable with scRNA-seq-specific methods such as ZINB-WaVE. For example, with the highest number of low-dimensional components, the average NMI across all data sets for FA, ICA, PCA, ZINB-WaVE, LLE, and MDS are 0.85, 0.84, 0.83, 0.83, 0.82, and 0.82, respectively. Their performance is followed by ZIFA (0.79), NMF (0.73), and DCA (0.69). The same four methods, pCMF (0.55), Poisson NMF (0.31), scScope (0.31), and LTSA (0.06) again do not fare well with a large number of low-dimensional components (Additional file 1: Figure S16A). The comparable results of generic dimensionality reduction methods with scRNA-seq-specific dimensionality reduction methods with a high number of low-dimensional components are also consistent some of the previous observations; for example, the original ZINB-WaVE paper observed that PCA can generally yield comparable results with scRNA-seq-specific dimensionality reduction methods in real data [32].

Besides the *k*-means clustering algorithm, we also used the hierarchical clustering algorithm to evaluate the performance of different dimensionality reduction methods (Additional file 1: Figure S17-S19). In this comparison, we had to exclude one dimensionality reduction method, scScope, as hierarchical clustering does not work on the extracted low-dimensional components from scScope. Consistent with the *k*-means clustering results, we found that the clustering accuracy measured by hierarchical clustering is relatively low when the number of low-dimensional components is very small (e.g., 2 or 0.5%), but generally increases with the number of included components. In addition, consistent with the *k*-means clustering results, we found that generic dimensionality reduction methods often yield results comparable to or better than scRNA-seq-specific dimensionality reduction methods (Additional file 1: Figure S17-S19). In particular, with a low number of low-dimensional components, MDS achieves the best performance (Additional file 1: Figure S19). With a moderate or high number of low-dimensional components, two generic dimensionality reduction methods, FA and NMF, often outperform various other dimensionality reduction methods across a range of settings. For example, when the number of low-dimensional components is moderate (6 or 1%), both FA and NMF achieve an average NMI value of 0.80 across data sets (Additional file 1: Figure S19A). In this case, their performance is followed by PCA (0.72), Poisson NMF (0.71), ZINB-WaVE (0.71), Diffusion Map (0.70), LLE (0.70), ICA (0.69), ZIFA (0.68), pCMF (0.65), and DCA (0.63). tSNE (0.31) does not fare well, either because it only extracts two-dimensional components or because it does not pair well with hierarchical clustering. We note, however, that the clustering results obtained by hierarchical clustering are often slightly worse than that obtained by *k*-means clustering across settings (e.g., Additional file 1: Figure S16 vs Additional file 1: Figure S19), consistent with the fact that many scRNA-seq clustering methods use *k*-means as a key ingredient [18, 25].

Finally, besides the *k*-means and hierarchical clustering methods, we also performed clustering analysis based on a community detection algorithm Louvain clustering method [61]. Unlike the *k*-means and hierarchical clustering methods, Louvain method does not require a pre-defined number of clusters and can infer the number of clusters in an automatic fashion. Following software recommendation [28, 61], we set the *k*-nearest neighbor parameter in Louvain method to be 50 for graph building in the analysis. We measured dimensionality reduction performance again by either average NMI (Additional file 1: Figure S20) or ARI (Additional file 1: Figure S21). Consistent with the *k*-means clustering results, we found that the clustering accuracy measured by Louvain method is relatively low when the number of low-dimensional components is very small (e.g., 2 or 0.5%), but generally increases with the number of included components. With a low number of low-dimensional components, ZINB-WaVE (0.72) achieves the best performance (Additional file 1: Figure S20-S22). With a moderate or high number of low-dimensional components, two generic dimensionality reduction methods, FA and MDS, often outperform various other dimensionality reduction methods across a range of settings (Additional file 1: Figure S20-S22). For example, when the number of low-dimensional components is high (6 or 1%), FA achieves an average NMI value of 0.77 across data sets (Additional file 1: Figure S22A). In this case, its performance is followed by NMF (0.76), MDS (0.75), GLMPCA (0.74), LLE (0.74), PCA (0.73), ICA (0.73), ZIFA (0.72), and ZINB-WaVE (0.72). Again consistent with the *k*-means clustering results, scScope (0.32) and LTSA (0.21) do not fare well. We also note that the clustering results obtained by Louvain method are often slightly worse than that obtained by *k*-means clustering and slightly better than that obtained by hierarchical clustering across settings (e.g., Additional file 1: Figure S16 vs Additional file 1: Figure S19 vs Additional file 1: Figure S22).

#### Normalization does not influence the performance of dimensionality reduction methods

While some dimensionality reduction methods (e.g., Poisson NMF, ZINB-WaVE, pCMF, and DCA) directly model count data, many dimensionality reduction methods (e.g., PCA, ICA, FA, NMF, MDS, LLE, LTSA, Isomap, Diffusion Map, UMAP, and tSNE) require normalized data. The performance of dimensionality reduction methods that use normalized data may depend on how data are normalized. Therefore, we investigated how different normalization approaches impact on the performance of the aforementioned dimensionality reduction methods that use normalized data. We examined two alternative data transformation approaches, log2 CPM (count per million; 11 dimensionality reduction methods), and *z*-score (10 dimensionality reduction methods), in addition to the log2 count we used in the previous results (transformation details are provided in “Methods and Materials”). The evaluation results are summarized in Additional file 1: Figure S23-S30 and are generally insensitive to the transformation approach deployed. For example, with the *k*-means clustering algorithm, when the number of low-dimensional components is small (1%), PCA achieves an NMI value of 0.82, 0.82, and 0.81, for log2 count transformation, log2 CPM transformation, and *z*-score transformation, respectively (Additional file 1: Figure S16A, S26A, and S30A). Similar results hold for the hierarchical clustering algorithm (Additional file 1: Figure S16B, S26B, and S30B) and Louvain clustering method (Additional file 1: Figure S16C, S26C, and S30C). Therefore, different data transformation approaches do not appear to substantially influence the performance of dimensionality reduction methods.

#### Performance of dimensionality reduction methods in UMI vs non-UMI-based data sets

scRNA-seq data generated from UMI-based technologies (e.g., 10X Genomics) are often of large scale, come with almost no amplification bias, do not display apparent dropout events, and can be accounted for by over-dispersed Poisson distributions. In contrast, data generated from non-UMI-based techniques (e.g., Smart-Seq2) are often of small scale, have high capture rate, and come with excessive dropout events. Subsequently, the unwanted variation from these two types of dataset can be quite different. To investigate how different dimensionality reduction methods perform in these two different types of data sets, we grouped 14 cell clustering data sets into a UMI-based group (7 data sets) and a non-UMI-based group (7 data sets). In the UMI-based data sets, we found that many dimensionality reduction methods perform reasonably well and their performance is relatively stable across a range of included low-dimensional components (Additional file 1: Figure S31A). For example, with the lowest number of low-dimensional components, the average NMI of PCA, ICA, FA, NMF, GLMPCA, ZINB-WaVE, and MDS are 0.73, 0.73, 0.73, 0.73, 0.74, and 0.75, respectively. Their performance remains similar with increasing number of low-dimensional components. However, a few dimensionality reduction methods, including Poisson NMF, pCMF, scScope, and LTSA, all have extremely low performance across settings. In the non-UMI-based data sets, the same set of dimensionality reduction methods perform reasonably well though their performance can vary with respect to the number of low-dimensional components (Additional file 1: Figure S31B). For example, with a low number of low-dimensional components, five dimensionality reduction methods, MDS, UMAP, ZINB-WaVE, ICA, and tSNE, perform reasonably well. The average NMI of these methods are 0.83, 0.81, 0.80, 0.78, and 0.77, respectively. With increasing number of low-dimensional components, four additional dimensionality reduction methods, PCA, ICA, FA, and ZINB-WaVE, also start to catch up. However, a similar set of dimensionality reduction methods, including GLMPCA, Poisson NMF, scScope, LTSA, and occasionally pCMF, also do not perform well in these non-UMI data sets.

#### Visualization of clustering results

We visualized the cell clustering results in two example data sets: the Kumar data which is non-UMI based and the PBMC3k data which is UMI based. The Kumar data consists of mouse embryonic stem cells cultured in three different media while the PBMC3k data consists of 11 blood cell types (data details in the Additional file 1). Here, we extracted 20 low-dimensional components in the Kumar data and 32 low low-dimensional components in the PBMC3k data with different dimensionality reduction methods. We then performed tSNE analysis on these low-dimensional components to extract the two tSNE components for visualization (Additional file 1: Figure S32-S33). Importantly, we found that the tSNE visualization results are not always consistent with clustering performance for different dimensionality reduction methods. For example, in the Kumar data, the low-dimensional space constructed by FA, pCMF, and MDS often yield clear clustering visualization with distinguish clusters (Additional file 1: Figure S32), consistent with their good performance in clustering (Fig. 2). However, the low-dimensional space constructed by PCA, ICA, and ZIFA often do not yield clear clustering visualization (Additional file 1: Figure S32), even though these methods all achieve high cell clustering performance (Fig. 2). Similarly, in the PBMC3k data set, FA and MDS perform well in clustering visualization (Additional file 1: Figure S33), which is consistent with their good performance in clustering analysis (Fig. 2). However, PCA and ICA do not fare well in clustering visualization (Additional file 1: Figure S33), even though both of them achieve high clustering performance (Fig. 2). The inconsistency between cluster visualization and clustering performance highlights the difference in the analytic goal of these two analyses: cluster visualization emphasizes on extracting as much information as possible using only the top two-dimensional components, while clustering analysis often requires a much larger number of low-dimensional components to achieve accurate performance. Subsequently, dimensionality reduction methods for data visualization may not fare well for cell clustering, and dimensionality reduction methods for cell clustering may not fare well for data visualization [20].

#### Rare cell type identification

So far, we have focused on clustering performance in terms of assigning all cells to cell types without distinguishing whether the cells belong to a rare population or a non-rare population. Identifying rare cell populations can be of significant interest in certain applications and performance of rare cell type identification may not always be in line with general clustering performance [62, 63]. Here, we examine the effectiveness of different dimensionality reduction methods in facilitating the detection of rare cell populations. To do so, we focused on the PBMC3k data from 10X Genomics [33]. The PBMC3k data were measured on 3205 cells with 11 cell types. We considered CD34+ cell type (17 cells) as the rare cell population. We paired the rare cell population with either CD19+ B cells (406 cells) or CD4+/CD25 T Reg cells (198) cells to construct two data sets with different rare cell proportions. We named these two data sets PBMC3k1Rare1 and PBMC3k1Rare2, respectively. We then applied different dimensionality reduction methods to each data and used *F*-measure to measure the performance of rare cell type detection following [64, 65] (details in “Methods and Materials”). The results are summarized in Additional file 1: Figure S34-S35.

Overall, we found that Isomap achieves the best performance for rare cell type detection across a range of low-dimensional components in both data sets with different rare cell type proportions. As expected, the ability to detect rare cell population increases with increasing rare cell proportions. In the PBMC3k1Rare1 data, the *F*-measure by Isomap with four different number of low-dimensional components (0.5%, 1%, 2%, and 3%) are 0.74, 0.79, 0.79, and 0.79, respectively (Additional file 1: Figure S34). The performance of Isomap is followed by ZIFA (0.74, 0.74, 0.74, and 0.74) and GLMPCA (0.74, 0.74, 0.73, and 0.74). In the PBMC3k1Rare2 data, the F-measure by Isomap with four different numbers of low-dimensional components (0.5%, 1%, 2%, and 3%) are 0.79, 0.79, 0.79, and 0.79, respectively (Additional file 1: Figure S35). The performance of Isomap is also followed by ZIFA (0.74, 0.74, 0.74, and 0.74) and GLMPCA (0.74, 0.74, 0.74, and 0.74). Among the remaining methods, Poisson NMF, pCMF, scScope, and LTSA do not fare well for rare cell type detection. We note that many dimensionality reduction methods in conjunction with Louvain clustering method often yield an *F*-measure of zero when the rare cell type proportion is low (Additional file 1: Figure S34C; PBMC3kRare1, 4.0% CD34+ cells) and only become reasonable with increasingly large rare cell type proportions (Additional file 1: Figure S35C; PBMC3kRare2, 7.9% CD34+ cells). The poor performance of the Louvain clustering method for rare cell type detection is likely because its automatic way of determining cell cluster number does not fare well in the presence of uneven/un-balanced cell type proportions.

#### Stability analysis across data splits

Finally, we investigated the stability and robustness of different dimensionality reduction methods. To do so, we randomly split the *Kumar* data into two subsets with an equal number of cells for each cell type in the two subsets. We applied each dimensionality reduction method to the two subsets and measured the clustering performance in each subset separately. We repeated the procedure 10 times to capture the potential stochasticity during the data split. We visualized the clustering performance of different dimensionality reduction methods in the two subsets separately. Such visualization allows us to check the effectiveness of dimensionality reduction methods with respect to reduced sample size in the subset, as well as the stability/variability of dimensionality reduction methods across different split replicates (Additional file 1: Figure S36). The results show that six dimensionality reduction methods, PCA, ICA, FA, ZINB-WaVE, MDS, and UMAP, often achieve both accurate clustering performance and highly stable and consistent results across the subsets. The accurate and stable performance of ICA, ZINB-WaVE, MDS, and UMAP is notable even with a relatively small number of low-dimensional components. For example, with very small number of low-dimensional components, ICA, ZINB-WaVE, MDS, and UMAP achieve an average NMI value of 0.98 across the two subsets, with virtually no performance variability across data splits (Additional file 1: Figure S36).

Overall, the results suggest that, in terms of downstream clustering analysis accuracy and stability, PCA, FA, NMF, and ICA are preferable across a range of data sets examined here. In addition, scRNA-seq-specific dimensionality reduction methods such as ZINB-WaVE, GLMPCA, and UMAP are also preferable if one is interested in extracting a small number of low-dimensional components, while generic methods such as PCA or FA are also preferred when one is interested in extracting a large number of low-dimensional components.

### Performance of dimensionality reduction methods for trajectory inference

**Fig. 3:**
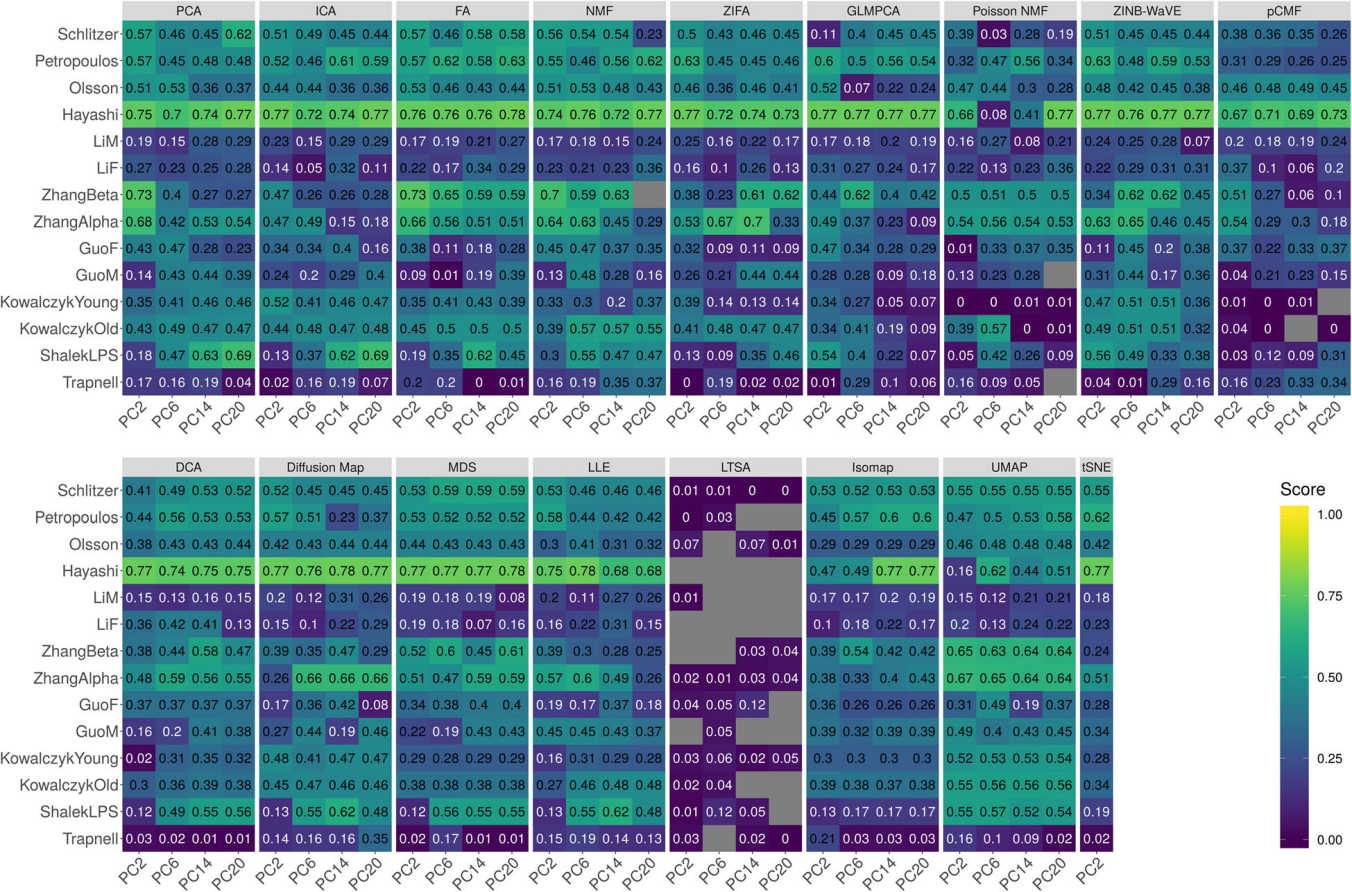
Dimensionality reduction method performance evaluated by Kendall correlation in the downstream trajectory inference analysis. We compared 17 dimensionality reduction methods (columns), including factor analysis (FA), principal component analysis (PCA), independent component analysis (ICA), Diffusion Map, nonnegative matrix factorization (NMF), Poisson NMF, zero-inflated factor analysis (ZIFA), zero-inflated negative binomial-based wanted variation extraction (ZINB-WaVE), probabilistic count matrix factorization (pCMF), deep count autoencoder network (DCA), generalized linear model principal component analysis (GLMPCA), multidimensional scaling (MDS), locally linear embedding (LLE), local tangent space alignment (LTSA), Isomap, uniform manifold approximation and projection (UMAP), and *t*-distributed stochastic neighbor embedding (tSNE). We evaluated their performance on 14 real scRNA-seq data sets (rows) in terms of lineage inference accuracy. We used *Slingshot* with *k*-means as the initial step for lineage inference. The performance of each dimensionality reduction method is measured by Kendall correlation. For each data set, we compared four different numbers of low-dimensional components (2, 6, 14, and 20; four sub-columns under each column). Gray fills in the table represents missing results where *Slingshot* gave out errors when we supplied the extracted low-dimensional components from the corresponding dimensionality reduction method. Note that, for tSNE, we only extracted two low-dimensional components due to the limitation of the tSNE software

We evaluated the performance of different dimensionality reduction methods for lineage inference applications (details in “Methods and Materials”). To do so, we obtained 14 publicly available scRNA-seq data sets, each of which contains known lineage information (Additional file 1: Table S2). The known lineages in all these data are linear, without bifurcation or multifurcation patterns. For each data set, we applied one dimensionality reduction method at a time to extract a fixed number of low-dimensional components. In the process, we varied the number of low-dimensional components from 2, 6, 14, to 20 to examine their influence for downstream analysis. With the extracted low-dimensional components, we applied two commonly used trajectory inference methods: *Slingshot* [66] and *Monocle3* [28, 67]. Slingshot is a clustering-dependent trajectory inference method, which requires additional cell label information. We therefore first used either *k*-means clustering algorithm, hierarchical clustering, or Louvain method to obtain cell type labels, where the number of cell types in the clustering was set to be the known truth. Afterwards, we supplied the low-dimensional components and cell type labels to the Slingshot to infer the lineage. Monocle3 is a clustering free trajectory inference method, which only requires low-dimensional components and trajectory starting state as inputs. We set the trajectory starting state as the known truth for Monocle3. Following [66], we evaluated the performance of dimensionality reduction methods by Kendall correlation coefficient (details in “Methods and Materials”) that compares the true lineage and inferred lineage obtained based on the low-dimensional components. In this comparison, we also excluded one dimensionality reduction method, scScope, which is not compatible with *Slingshot*. The lineage inference results for the remaining dimensionality reduction methods are summarized in Fig. 3 and Additional file 1: Figure S37-S54.

#### Trajectory inference by Slingshot

We first focused on the comparison results obtained from Slingshot. Different from the clustering results where accuracy generally increases with increasing number of included low-dimensional components, the lineage tracing results from Slingshot do not show a clear increasing pattern with respect to the number of low-dimensional components, especially when we used *k*-means clustering as the initial step (Fig. 3 and Additional file 1: Figure S39A). For example, the average Kendall correlations across all data sets and across all methods are 0.35, 0.36, 0.37, and 0.37 for increasingly large number of components, respectively. When we used hierarchical clustering algorithm as the initial step, the lineage tracing results in the case of a small number of low-dimensional components are slightly inferior as compared to the results obtained using a large number of low-dimensional components (Additional file 1: Figure S37 and S39B). However, we do note that the lineage tracing results obtained using *k*-means are better than that obtained using hierarchical clustering as the initial step. In addition, perhaps somewhat surprisingly, the lineage tracing results obtained using Louvain clustering method are slightly better that the results obtained using *k*-means clustering (Additional file 1: Figure S38 and S39C)—even though the clustering results from *k*-means are generally better than that from Louvain. For example, the average Kendall correlations obtained using Louvain method across all data sets and across all methods are 0.36, 0.38, 0.40, and 0.40 for increasingly large number of components, respectively. Therefore, Louvain method is recommended as the initial step for lineage inference and a small number of low-dimensional components there is often sufficient for accurate results. When conducting lineage inference based on a low number of components with Louvain method, we found that four dimensionality reduction methods, PCA, FA, ZINB-WaVE, and UMAP, all perform well for lineage inference across varying number of low-dimension components (Additional file 1: Figure S39C). For example, with the lowest number of components, the average Kendall correlations across data sets for PCA, FA, UMAP, and ZINB-WaVE are 0.44, 0.43, 0.40, and 0.43, respectively. Their performance is followed by ICA (0.37), ZIFA (0.36), tSNE (0.33), and Diffusion Map (0.38), while pCMF (0.26), Poisson NMF (0.26), and LTSA (0.12) do not fare well.

#### Trajectory inference by Monocle3

We next examined the comparison results based on Monocle3 (Additional file 1: Figure S40-S41). Similar to Slingshot, we found that the lineage tracing results from Monocle3 also do not show a clear increasing pattern with respect to the number of low-dimensional components (Additional file 1: Figure S41). For example, the average Kendall correlations across all data sets and across all methods are 0.37, 0.37, 0.38, and 0.37 for an increasingly large number of components, respectively. Therefore, similar with Slingshot, we also recommend the use of a small number of low-dimensional components with Monocle3. In terms of dimensionality reduction method performance, we found that five dimensionality reduction methods, FA, MDS, GLMPCA, ZINB-WaVE, and UMAP, all perform well for lineage inference. Their performance is often followed by NMF and DCA, while Poisson NMF, pCMF, LLE, and LTSA do not fare well. The dimensionality reduction comparison results based on Monocle3 are in line with those recommendations by Monocle3 software, which uses UMAP as the default dimensionality reduction method [28]. In addition, the set of five top dimensionality reduction methods for Monocle3 are largely consistent with the set of top five dimensionality reduction methods for Slingshot, with only one method difference between the two (GLMPCA in place of PCA). The similarity of top dimensionality reduction methods based on different lineage inference methods suggests that a similar set of dimensionality reduction methods are likely suitable for lineage inference in general.

#### Visualization of inferred lineages

We visualized the reduced low-dimensional components from different dimensionality reduction methods in one trajectory data set, the ZhangBeta data. The ZhangBeta data consists of expression measurements on mouse pancreatic *β* cells collected at seven different developmental stages. These seven different cell stages include E17.5, P0, P3, P9, P15, P18, and P60. We applied different dimensionality reduction methods to the data to extract the first two-dimensional components. Afterwards, we performed lineage inference and visualization using Monocle3. The inferred tracking paths are shown in Additional file 1: Figure S42. Consistent with Kendall correlation (Fig. 3), all top dimensionality reduction methods are able to infer the correct lineage path. For example, the trajectory from GLMPCA and UMAP completely matches the truth. The trajectory inferred from FA, NMF, or ZINB-WaVE largely matches the truth with small bifurcations. In contrast, the trajectory inferred from either Poisson NMF or LTSA displays unexpected radical patterns (Additional file 1: Figure S42), again consistent with the poor performance of these two methods in lineage inference.

#### Normalization does not influence the performance of dimensionality reduction methods

For dimensionality reduction methods that require normalized data, we further examined the influence of different data transformation approaches on their performance (Additional file 1: Figure S43-S53). Like in the clustering comparison, we found that different transformations do not influence the performance results for most dimensionality reduction methods in lineage inference. For example, in Slingshot with the *k*-means clustering algorithm as the initial step, when the number of low-dimensional components is small, UMAP achieves a Kendall correlation of 0.42, 0.43, and 0.40, for log2 count transformation, log2 CPM transformation, and *z*-score transformation, respectively (Additional file 1: Figure S39A, S46A, and S50A). Similar results hold for the hierarchical clustering algorithm (Additional file 1: Figure S39B, S46B, and S50B) and Louvain method (Additional file 1: Figure S39B, S46B, and S50B). However, some notable exceptions exist. For example, with log2 CPM transformation but not the other transformations, the performance of Diffusion Map increases with increasing number of included components when *k*-means clustering was used as the initial step: the average Kendall correlations across different low-dimensional components are 0.37, 0.42, 0.44, and 0.47, respectively (Additional file 1: Figure S43 and S46A). As another example, with *z*-score transformation but not with the other transformations, FA achieves the highest performance among all dimensionality reduction methods across different number of low-dimensional components (Additional file 1: Figure S50A). Similarly, in Monocle3, different transformations (log2 count transformation, log2 CPM transformation, and *z*-score transformation) do not influence the performance of dimensionality reduction methods. For example, with the lowest number of low-dimensional components, UMAP achieves a Kendall correlation of 0.49, 0.47, and 0.47, for log2 count transformation, log2 CPM transformation, and *z*-score transformation, respectively (Additional file 1: Figure S41, S53A, and S53B).

#### Stability analysis across data splits

We also investigated the stability and robustness of different dimensionality reduction methods by data split in the *Hayashi* data. We applied each dimensionality reduction method to the two subsets and measured the lineage inference performance in the two subsets separately. We again visualized the clustering performance of different dimensionality reduction methods in the two subsets, separately. Such visualization allows us to check the effectiveness of dimensionality reduction methods with respective to reduced sample size in the subset, as well as the stability/variability of dimensionality reduction methods across different split replicates (Additional file 1: Figure S54). The results show that four of the dimensionality reduction methods, FA, Diffusion Map, ZINB-WaVE, and MDS often achieve both accurate performance and highly stable and consistent results across the subsets. The accurate and stable performance of these is notable even with a relatively small number of low-dimensional components. For example, with a very small number of low-dimensional components, FA, Diffusion Map, ZINB-WaVE, and MDS achieve a Kendall correlation of 0.75, 0.77, 0.77, and 0.78 averaged across the two subsets, respectively, and again with virtually no performance variability across data splits (Additional file 1: Figure S54).

Overall, the results suggest that, in terms of downstream lineage inference accuracy and stability, the scRNA-seq non-specific dimensionality reduction method FA, PCA, and NMF are preferable across a range of data sets examined here. The scRNA-seq-specific dimensionality reduction methods ZINB-WaVE as well as the scRNA-seq non-specific dimensionality reduction method NMF are also preferable if one is interested in extracting a small number of low-dimensional components for lineage inference. In addition, the scRNA-seq-specific dimensionality reduction method Diffusion Map and scRNA-seq non-specific dimensionality reduction method MDS may also be preferable if one is interested in extracting a large number of low-dimensional components for lineage inference.

### Large-scale scRNA-seq data applications

Finally, we evaluated the performance of different dimensionality reduction methods in two large-scale scRNA-seq data sets. The first data is Guo et al. [68], which consists of 12,346 single cells collected through a non-UMI-based sequencing technique. Guo et al. data contains known cell cluster information and is thus used for dimensionality reduction method comparison based on cell clustering analysis. The second data is Cao et al. [28], which consists of approximately 2 million single cells collected through a UMI-based sequencing technique. Cao et al. data contains known lineage information and is thus used for dimensionality reduction method comparison based on trajectory inference. Since many dimensionality reduction methods are not scalable to these large-scale data sets, in addition to applying dimensionality reduction methods to the two data directly, we also coupled them with a recently developed sub-sampling procedure *dropClust* to make all dimensionality reduction methods applicable to large data [69] (details in “Methods and Materials”). We focus our comparison in the large-scale data using the *k*-means clustering method. We also used log2 count transformation for dimensionality reduction methods that require normalized data.

The comparison results when we directly applied dimensionality reduction methods to the Guo et al. data are shown in Additional file 1: Figure S55. Among the methods that are directly applicable to large-scale data sets, we found that UMAP consistently outperforms the remaining dimensionality reduction methods across a range of low-dimensional components by a large margin. For example, the average NMI of UMAP across different number of low-dimensional components (0.5%, 1%, 2%, and 3%) are in the range between 0.60 and 0.61 (Additional file 1: Figure S55A). In contrast, the average NMI for the other methods are in the range of 0.15–0.51. In the case of a small number of low-dimensional components, we found that the performance of both FA and NMF are reasonable and follow right after UMAP. With the sub-sampling procedure, we can scale all dimensionality reduction methods relatively easily to this large-scale data (Additional file 1: Figure S56). As a result, several dimensionality reduction methods, most notably FA, can achieve similar or better performance as compared to UMAP. However, we do notice an appreciable performance loss for many dimensionality reduction methods through the sub-sampling procedure. For example, the NMI of UMAP in the sub-sampling-based procedure is only 0.26, representing an approximately 56% performance loss compared to the direct application of UMAP without sub-sampling (Additional file 1: Figure S56 vs Figure S55). Therefore, we caution the use of sub-sampling procedure and recommend users to careful examine the performance of dimensionality reduction methods before and after sub-sampling to decide whether sub-sampling procedure is acceptable for their own applications.

For lineage inference in the Cao et al. data, due to computational constraint, we randomly obtained 10,000 cells from each of the five different developmental stages (i.e., E9.5, E10.5, E11.5, E12.5, and E13.5) and applied different dimensionality reduction methods to analyze the final set of 50,000 cells. Because most dimensionality reduction methods are not scalable even to these 50,000 cells, we only examined the performance of dimensionality reduction methods when paired with the sub-sampling procedure (Additional file 1: Figure S57). With the small number of low-dimensional components, three dimensionality reduction methods, GLMPCA, DCA, and Isomap, all achieve better performance than the other dimensionality reduction methods. For example, with the lowest number of low-dimensional components, the average absolute Kendall correlations of GLMPCA, DCA, and Isomap are 0.13, 0.28, and 0.17, respectively. In contrast, the average absolute Kendall correlations of the other dimensionality reduction methods are in the range of 0.01–0.12. With a higher number of low-dimensional components, Isomap and UMAP show better performance. For example, with 3% low-dimensional components, the average absolute Kendall correlations of Isomap and UMAP increase to 0.17 and 0.30, respectively. Their performance is followed by Diffusion Map (0.15), ZINB-WaVE (0.14), and LLE (0.12), while the remaining methods are in the range of 0.04–0.07.

### Computation time

**Fig. 4:**
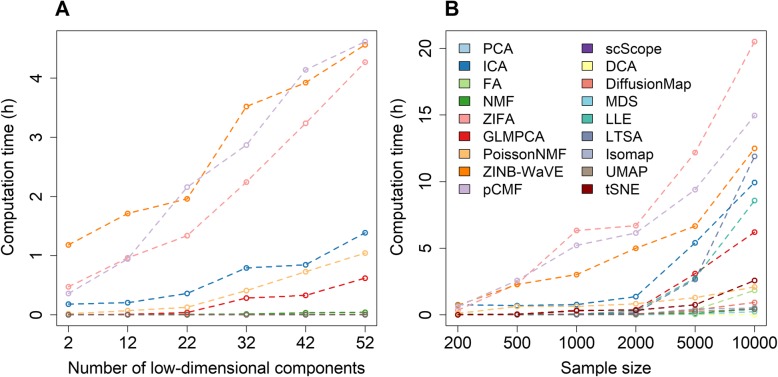
The computation time (in hours) for different dimensionality reduction methods. We recorded computing time for 18 dimensionality reduction methods on simulated data sets with a varying number of low-dimensional components and a varying number of sample sizes. Compared dimensionality reduction methods include factor analysis (FA; light green), principal component analysis (PCA; light blue), independent component analysis (ICA; blue), Diffusion Map (pink), nonnegative matrix factorization (NMF; green), Poisson NMF(light orange), zero-inflated factor analysis (ZIFA; light pink), zero-inflated negative binomial based wanted variation extraction (ZINB-WaVE; orange), probabilistic count matrix factorization (pCMF; light purple), deep count autoencoder network (DCA; yellow), scScope (purple), generalized linear model principal component analysis (GLMPCA; red), multidimensional scaling (MDS; cyan), locally linear embedding (LLE; blue green), local tangent space alignment (LTSA; teal blue), Isomap (gray), uniform manifold approximation and projection (UMAP; brown), and *t*-distributed stochastic neighbor embedding (tSNE; dark red). **a** Computation time for different dimensionality reduction methods (*y*-axis) changes with respect to an increasing number of low-dimensional components (*x*-axis). The number of cells is fixed to be 500 and the number of genes is fixed to be 10,000 in this set of simulations. Three methods (ZINB-WaVE, pCMF, and ZIFA) become noticeably computationally more expensive than the remaining methods with increasing number of low-dimensional components. **b** Computation time for different dimensionality reduction methods (*y*-axis) changes with respect to an increasing sample size (i.e., the number of cells) in the data. Computing time is recorded on a single thread of an Intel Xeon E5-2683 2.00-GHz processor. The number of low-dimensional components is fixed to be 22 in this set of simulations for most methods, except for tSNE which used two low-dimensional components due to the limitation of the tSNE software. Note that some methods are implemented with parallelization capability (e.g., ZINB-WaVE and pCMF) though we tested them on a single thread for fair comparison across methods. Note that PCA is similar to ICA in **a** and scScope is similar to several other efficient methods in **b**; thus, their lines may appear to be missing. Overall, three methods (ZIFA, pCMF, and ZINB-WaVE) become noticeably computationally more expensive than the remaining methods with increasing number of cells in the data

We recorded and compared computing time for different dimensionality reduction methods on simulated data sets. Here, we also examined how computation time for different dimensionality reduction methods varies with respect to the number of low-dimensional components extracted (Fig. 4a) as well as with respect to the number of cells contained in the data (Fig. 4b). Overall, the computational cost of three methods, ZINB-WaVE, ZIFA, and pCMF, is substantially heavier than that of the remaining methods. Their computation time increases substantially with both increasingly large number of low-dimensional components and increasingly large number of cells in the data. Specifically, when the sample size equals 500 and the desired number of low-dimensional components equals 22, the computing time for ZINB-WaVE, ZIFA, and pCMF to analyze 10,000 genes are 2.15, 1.33, and 1.95 h, respectively (Fig. 4a). When the sample size increases to 10,000, the computing time for ZINB-WaVE, ZIFA, and pCMF increases to 12.49, 20.50, and 15.95 h, respectively (Fig. 4b). Similarly, when the number of low-dimensional components increases to 52, the computing time for ZINB-WaVE, ZIFA, and pCMF increases to 4.56, 4.27, and 4.62 h, respectively. Besides these three methods, the computing cost of ICA, GLMPCA, and Poisson NMF can also increase noticeably with increasingly large number of low-dimensional components. The computing cost of ICA, but to a lesser extent of GLMPCA, LLE, LTSA, and Poisson NMF, also increases substantially with increasingly large number of cells. In contrast, PCA, FA, Diffusion Map, UMAP, and the two deep-learning-based methods (DCA and scScope) are computationally efficient. In particular, the computation times for these six methods are stable and do not show substantial dependence on the sample size or the number of low-dimensional components. Certainly, we expect that the computation time of all dimensionality reduction methods will further increase as the sample size of the scRNA-seq data sets increases in magnitude. Overall, in terms of computing time, PCA, FA, Diffusion Map, UMAP, DCA, and scScope are preferable.

### Practical guidelines

**Fig. 5:**
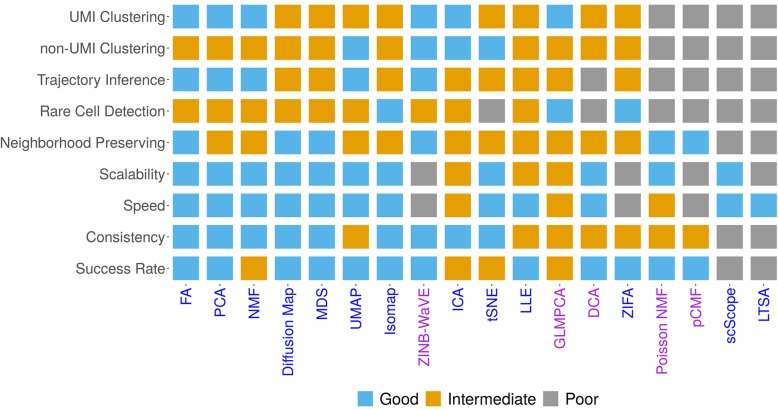
Practical guideline for choosing dimensionality reduction methods in scRNA-seq analysis. Compared dimensionality reduction methods include factor analysis (FA), principal component analysis (PCA), independent component analysis (ICA), Diffusion Map, nonnegative matrix factorization (NMF), Poisson NMF, zero-inflated factor analysis (ZIFA), zero-inflated negative binomial-based wanted variation extraction (ZINB-WaVE), probabilistic count matrix factorization (pCMF), deep count autoencoder network (DCA), scScope, generalized linear model principal component analysis (GLMPCA), multidimensional scaling (MDS), locally linear embedding (LLE), local tangent space alignment (LTSA), Isomap, uniform manifold approximation and projection (UMAP), and *t*-distributed stochastic neighbor embedding (tSNE). The count-based methods are colored in purple while non-count-based methods are colored in blue. Methods are ranked by their average performance across the criteria from left to right. The performance is colored and numerically coded: good performance = 2 (sky blue), intermediate performance = 1 (orange), and poor performance = 0 (gray)

In summary, our comparison analysis shows that different dimensionality reduction methods can have different merits for different tasks. Subsequently, it is not straightforward to identify a single dimensionality reduction method that strives the best in all data sets and for all downstream analyses. Instead, we provide a relatively comprehensive practical guideline for choosing dimensionality reduction methods in scRNA-seq analysis in Fig. 5. Our guideline is based on the accuracy and effectiveness of dimensionality reduction methods in terms of the downstream analysis, the robustness and stability of dimensionality reduction methods in terms of replicability and consistency across data splits, as well as their performance in large-scale data applications, data visualization, and computational scalability for large scRNA-seq data sets. Briefly, for cell clustering analysis, PCA, ICA, FA, NMF, and ZINB-WaVE are recommended for small data where computation is not a concern. PCA, ICA, FA, and NMF are also recommended for large data where computation is a concern. For lineage inference analysis, FA, PCA, NMF, UMAP, and ZINB-WaVE are all recommended for small data. A subset of these methods, FA, PCA, NMF, and UMAP are also recommended for large scRNA-seq data. In addition, for very large scRNA-seq data sets (e.g., > 100,000 samples), DCA and UMAP perhaps are the only feasible approach for both downstream analyses with UMAP being the preferred choice. We also recognize that PCA, ICA, FA, and NMF can be useful options in very large data sets when paired with a sub-sampling procedure [69], though care needs to be taken to examine the effectiveness of the sub-sampling procedure itself. Finally, besides these general recommendations, we note that some methods have additional features that are desirable for practitioners. For example, both ZINB-WaVE and GLMPCA can include sample-level and gene-level covariates, thus allowing us to easily control for batch effects or size factors. We provide our detailed recommendations in Fig. 5.

## Discussion

We have presented a comprehensive comparison of different dimensionality reduction methods for scRNA-seq analysis. We hope the summary of these state-of-the-art dimensionality reduction methods, the detailed comparison results, and the recommendations and guidelines for choosing dimensionality reduction methods can assist researchers in the analysis of their own scRNA-seq data.

In the present study, we have primarily focused on three clustering methods (*k*-means, hierarchical clustering, and Louvain method) to evaluate the performance of different dimensionality reduction methods for downstream clustering analysis. We have also primarily focused on two lineage inference methods (Slingshot and Monocle3) to evaluate the performance of different dimensionality reduction methods for downstream lineage inference. In our analysis, we found that the performance of dimensionality reduction methods measured based on different clustering methods is often consistent with each other. Similarly, the performance of dimensionality reduction methods measured based on different lineage inference methods is also consistent with each other. However, it is possible that some dimensionality reduction methods may work well with certain clustering approaches and/or with certain lineage inference approaches. Subsequently, future comparative analysis using other clustering methods and other lineage inference methods as comparison criteria may have added benefits. In addition, besides cell clustering and trajectory inference, we note that dimensionality reduction methods are also used for many other analytic tasks in scRNA-seq studies. For example, factor models for dimensionality reduction is an important modeling part for multiple scRNA-seq data set alignment [16], for integrative analysis of multiple omics data sets [70, 71], as well as for deconvoluting bulk RNA-seq data using cell type-specific gene expression measurements from scRNA-seq [72, 73]. In addition, cell classification in scRNA-seq also relies on a low-dimensional structure inferred from original scRNA-seq through dimensionality reduction [74, 75]. Therefore, the comparative results obtained from the present study can provide important insights into these different scRNA-seq analytic tasks. In addition, investigating the performance of dimensionality reduction methods in these different scRNA-seq downstream analyses is an important future research direction.

We mostly focused on evaluating feature extraction methods for dimensionality reduction. Another important category of dimensionality reduction method is the feature selection method, which aims to select a subset of features/genes directly from the original feature space. The feature section methods rely on different criteria to select important genes and are also commonly used in the preprocessing step of scRNA-seq data analysis [76]. For example, M3Drop relies on dropout events in scRNA-seq data to identify informative genes [77]. Seurat uses gene expression variance to select highly variable genes [16]. Evaluating the benefits of different methods and criteria for selecting informative genes for different downstream tasks is another important future direction.

We have primarily focused on using the default software settings when applying different dimensionality reduction methods. We note, however, that modifying the software setting for certain methods on certain data types may help improve performance. For example, a recent study shows that the quasi-UMI approach paired with GLMPCA may help improve the performance of GLMPCA on non-UMI data sets [78]. In addition, we have relied on a relatively simple gene filtering step by removing lowly expressed genes. Sophisticated gene filtering approaches prior to running dimensionality reduction may help improve the performance of certain dimensionality reduction methods. In addition, alternative, more stringent gene filtering approaches may likely result in a smaller subset of genes for performing dimensionality reduction, making it easier to apply some of the slow dimensionality reduction methods to large data sets. Exploring how different software settings and gene filtering procedures influence the performance of different dimensionality reduction methods on different data sets will help us better understand the utility of these methods.

With the advance of scRNA-seq technologies and with the increase collaborations across scientific groups, new consortium projects such as the Human Cell Atlas (HCA) will generate scRNA-seq data sets that contain millions of cells [34]. The large data at this scale poses critical computational and statistical challenges to many current dimensionality reduction methods. Many existing dimensionality reduction methods, in particular those that require the computation and memory storage of a covariance or distance matrix among cells, will no longer be applicable there. We have examined a particular sub-sampling strategy to scale all dimensionality reduction methods to large data sets. However, while the sub-sampling strategy is computationally efficient, it unfortunately reduces the performance of many dimensionality reduction methods by a substantial margin. Therefore, new algorithmic innovations and new efficient computational approximations will likely be needed to effectively scale many of the existing dimensionality reduction methods to millions of cells.

## Methods and materials

### ScRNA-seq data sets

We obtained a total of 30 scRNA-seq data sets from public domains for benchmarking dimensionality reduction methods. All data sets were retrieved from the Gene Expression Omnibus (GEO) database (https://www.ncbi.nlm.nih.gov/geo/) or the 10X Genomics website (https://support.10xgenomics.com/single-cell-gene-expression/datasets). These data sets cover a wide variety of sequencing techniques that include Smart-Seq2 (8 data sets), 10X Genomics (6 data sets), Smart-Seq (5 data sets), inDrop (1 data set), RamDA-seq (1 data set), sci-RNA-seq3 (1 data set), SMARTer (5 data sets), and others (3 data sets). In addition, these data cover a range of sample sizes from a couple hundred cells to tens of thousands of cells measured in either human (19 data sets) or mouse (11 data sets). In each data set, we evaluated the effectiveness of different dimensionality reduction methods for one of the two important downstream analysis tasks: cell clustering and lineage inference. In particular, 15 data sets were used for cell clustering evaluation while another 15 data sets were used for lineage inference evaluation. For cell clustering, we followed the same criteria listed in [12, 41] to select these datasets. In particular, the selected data sets need to contain true cell clustering information which is to be treated as the ground truth in the comparative analysis. In our case, 11 of the 15 data sets were obtained by mixing cells from different cell types either pre-determined by fluorescence activated cell sorting (FACS) or cultured on different conditions. Therefore, these 11 studies contain the *true* cell type labels for all cells. The remaining 4 data sets contain cell labels that were determined in the original study and we simply treated them as truth though we do acknowledge that such “true” clustering information may not be accurate. For lineage inference, we followed the same criteria listed in [14] to select these datasets. In particular, the selected data sets need to contain true linear lineage information which is to be treated as the ground truth in the comparative analysis. In our case, 4 of the 15 data sets were obtained by mixing cells from different cell types pre-determined by FACS. These different cell types are at different developmental stages of a single linear lineage; thus, these 4 studies contain the *true* lineage information for all cells. The remaining 11 data sets contain cells that were collected at multiple time points during the development process. For these data, we simply treated cells at these different time points as part of a single linear lineage, though we do acknowledge that different cells collected at the same time point may represent different developmental trajectories from an early time point if the cells at the early time are heterogeneous. In either case, the true lineages in all these 15 data sets are treated as linear, without any bifurcation or multifurcation patterns.

A detailed list of the selected scRNA-seq datasets with corresponding data features is provided in Additional file 1: Table S1-S2. In each of the above 30 data sets, we removed genes that are expressed in less than five cells. For methods modeling normalized data, we transformed the raw counts data into continuous data with the *normalize* function implemented in *scater* (R package v1.12.0). We then applied log2 transformation on the normalized counts by adding one to avoid log transforming zero values. We simply term this normalization as log2 count transformation, though we do acknowledge that such transformation does take into account of cell size factor, etc. through the *scater* software. In addition to log2 count transformation, we also explored the utility of two additional data transformation: log2 CPM transformation and *z*-score transformation. In the log2 CPM transformation, we first computed counts per million reads (CPM) and then performed log2 transformation on the resulted CPM value by adding a constant of one to avoid log transformation of zero quantities. In the *z*-score transformation, for each gene in turn, we standardized CPM values to achieve a mean of zero and variance of one across cells using *Seurat* package (v2.3).

Besides the above 30 real scRNA-seq data sets, we also simulated 2 additional scRNA-seq data sets for cell clustering evaluation. In the simulations, we used all 94 cells from one cell type (*v6.5 mouse 2i+LIF*) in the Kumar data as input. We simulated scRNA-seq data with 500 cells and a known number of cell types, which were set to be either 4 or 8, using the *Splatter* package v1.2.0. All parameters used in the *Splatter* (e.g., mean rate, shape, dropout rate) were set to be approximately those estimated from the real data. In the case of 4 cell types, we set the group parameter in *Splatter* as 4. We set the percentage of cells in each group as 0.1, 0.15, 0.5, and 0.25, respectively. We set the proportion of the differentially expressed genes in each group as 0.02, 0.03, 0.05, and 0.1, respectively. In the case of 8 cell types, we set group/cell type parameter as 8. We set the percentage of cells in each group as 0.12, 0.08, 0.1, 0.05, 0.3, 0.1, 0.2, and 0.05, respectively. We set the proportion of the differentially expressed genes in each group as 0.03, 0.03, 0.03, 0.1, 0.05, 0.07, 0.08, and 0.1, respectively.

### Compared dimensionality reduction methods

Dimensionality reduction methods aim to transform an originally high-dimensional feature space into a low-dimensional representation with a much-reduced number of components. These components are in the form of a linear or non-linear combination of the original features (known as feature extraction dimensionality reduction methods) [79] and in the extreme case are themselves a subset of the original features (known as feature selection dimensionality reduction methods) [80]. In the present study, we have collected and compiled a list of 18 popular and widely used dimensionality reduction methods in the field of scRNA-seq analysis. These dimensionality reduction methods include factor analysis (FA; R package *psych*, v1.8.12), principal component analysis (PCA; R package *stats*, v3.6.0), independent component analysis (ICA; R package *ica*, v1.0.2), Diffusion Map (Diffusion Map; R package *destiny*, v2.14.0), nonnegative matrix factorization (NMF; R package NNLM, v1.0.0), Kullback-Leibler divergence-based NMF (Poisson NMF; R package NNLM, v1.0.0), zero-inflated factor analysis (ZIFA; Python package *ZIFA*), zero-inflated negative binomial-based wanted variation extraction (ZINB-WaVE; R package *zinbwave*, v1.6.0), probabilistic count matrix factorization (pCMF; R package pCMF, v1.0.0), deep count autoencoder network (DCA; Python package *dca*), a scalable deep-learning-based approach (scScope; Python package *scscope*), generalized linear model principal component analysis (GLMPCA; R package on github), multidimensional scaling (MDS; Rdimtools R package v.0.4.2), locally linear embedding (LLE; Rdimtools R packge v.0.4.2), local tangent space alignment (LTSA; Rdimtools R package v.0.4.2), Isomap (Rdimtools R package v.0.4.2), t-distributed stochastic neighbor embedding (tSNE; FIt-SNE, *fftRtnse* R function), and uniform manifold approximation and projection (UMAP; Python package). One of these methods, tSNE, can only extract a maximum of two or three low-dimensional components [48, 58, 59]. Therefore, we only included tSNE results based on two low-dimensional components extracted from the recently developed fast *FIt-SNE* R package [48] in all figures. An overview of these 18 dimensionality reduction methods with their corresponding modeling characteristics is provided in Table 1.

### Assess the performance of dimensionality reduction methods

We first evaluated the performance of dimensionality reduction methods by neighborhood preserving that aims to access whether the reduced dimensional space resembles the original gene expression matrix. To do so, we first identified the *k*-nearest neighbors for each single cell in the original space (denoted as a set A) and in the reduced space (denoted as a set B). We set *k* = 10, 20, or 30 in our study. We then computed the Jaccard index (JI) [60] to measure the neighborhood similarity between the original space and the reduced space: $JI=\frac{\left|A\cap B\right|}{\left|A\cup B\right|}$, where |∙| denotes the cardinality of a set. We finally obtained the averaged Jaccard index (AJI) across all cells to serve as the measurement for neighborhood preserving. We note, however, that neighborhood preserving is primarily used to measure the effectiveness of pure dimensionality reduction in terms of preserving the original space and may not be relevant for single-cell analytic tasks that are the main focus of the present study: a dimensionality reduction method that preserve the original gene expression matrix effectively may not be effective in extracting useful biological information from the expression matrix that are essential for key downstream single-cell applications. Preserving the original gene expression matrix is rarely the purpose of dimensionality reduction methods for single-cell applications: indeed, the original gene expression matrix (which is the best-preserved matrix of itself) is rarely, if ever, used directly in any downstream single-cell applications including cell clustering and lineage inference, even though it is computationally easy to do so.

Therefore, more importantly, we also evaluated the performance of dimensionality reduction methods by evaluating how effective the low-dimensional components extracted from dimensionality reduction methods are for downstream single-cell analysis. We evaluated either of the two commonly applied downstream analysis, clustering analysis, and lineage reconstruction analysis, in the 32 data sets described above. In the analysis, we varied the number of low-dimensional components extracted from these dimensionality reduction methods. Specifically, for cell clustering data sets, in a data with less than or equal to 300 cells, we varied the number of low-dimensional components to be either 2, 6, 14, or 20. In a data with more than 300 cells, we varied the number of low-dimensional components to be either 0.5%, 1%, 2%, or 3% of the total number of cells. For lineage inference data sets, we varied the number of low-dimensional components to be either 2, 6, 14, or 20 for all data sets, since common lineage inference methods prefer a relatively small number of components.

For clustering analysis, after dimensionality reduction with these dimensionality reduction methods, we used three different clustering methods, the hierarchical clustering (R function *hclust*; stats v3.5.3), *k*-means clustering (R function *kmeans*; stats v3.6.0), or Louvain method (R function *clusterCells*; monocle v2.12.0) to perform clustering on the reduced feature space. The *k*-means clustering is a key ingredient of commonly applied scRNA-seq clustering methods such as SC3 [18] and Waterfall [25]. The hierarchical clustering is a key ingredient of commonly applied scRNA-seq clustering methods such as CIDR [17] and CHETAH [81]. The Louvain method is also a commonly used clustering method for common single-cell analysis software such as Seurat [16] and Monocle [27, 82]. In all these clustering methods, we set the number of clusters *k* to be the known number of cell types in the data. We compared the cell clusters inferred using the low-dimensional components to the true cell cluster and evaluated clustering accuracy by two criteria: the adjusted rand index (ARI) [83] and the normalized mutual information (NMI) [84]. The ARI and NMI are defined as:

$$ ARI\left(P,T\right)=\frac{\sum_{l,s}\left(\begin{array}{c}{n}_{ls}\\ {}2\end{array}\right)-\left[{\sum}_l\left(\begin{array}{c}{a}_l\\ {}2\end{array}\right){\sum}_s\left(\begin{array}{c}{b}_s\\ {}2\end{array}\right)\right]/\left(\begin{array}{c}n\\ {}2\end{array}\right)}{\frac{1}{2}\left[{\sum}_l\left(\begin{array}{c}{a}_l\\ {}2\end{array}\right)+{\sum}_s\left(\begin{array}{c}{b}_s\\ {}2\end{array}\right)\right]-\left[{\sum}_l\left(\begin{array}{c}{a}_l\\ {}2\end{array}\right){\sum}_s\left(\begin{array}{c}{b}_s\\ {}2\end{array}\right)\right]/\left(\begin{array}{c}n\\ {}2\end{array}\right)} $$ and $$ NMI\left(P,T\right)=\frac{2 MI\left(P,T\right)}{H(P)+H(T)}, $$

where *P* = (*p*_1_, *p*_2_, ⋯, *p*_*n*_)^*T*^ denotes the inferred cell type cluster labels from clustering analysis while *T* = (*t*_1_, *t*_2_, ⋯, *t*_*n*_)^*T*^ denotes the known true cell type labels for *n* samples in the data; *l* and *s* enumerate the clusters, with *l* = 1, ⋯, *r* and *s* = 1, ⋯, *k* where *r* and *k* are the number of inferred cell type clusters and the number of true cell type clusters, respectively; *n*_*ls*_ = ∑_*ij*_*I*(*p*_*i*_ = *l*)*I*(*t*_*j*_ = *s*) is the number of times where the *i*th cell belongs to the cluster *l* in the inferred cluster labeling and *j*th cell belongs to the cluster *s* in the true cluster labeling; note that *n*_*ls*_ is an entry of contingency table which effectively measures the number of cells that are in common between *P* and *T*, with *I*(∙) being an indicator function; *a*_*l*_ = ∑_*s*_*n*_*ls*_ is the sum of the *s*th column of the contingency table; and *b*_*s*_ = ∑_*l*_*n*_*ls*_ is the sum of the *l*th row of the contingency table; $$ \left(\begin{array}{c}\bullet \\ {}\bullet \end{array}\right) $$ denotes a binomial coefficient; $MI\left(P,T\right)={\sum}_l{\sum}_s\frac{n_{ls}}{n}\mathit{\log}\left(\frac{\frac{n_{ls}}{n}}{\frac{b_s{a}_l}{n^2}}\right)$ is the mutual information between two cluster labels; $H(P)=-{\sum}_l\frac{a_l}{n}\mathit{\log}\left(\frac{a_l}{n}\right)$ is the entropy function for inferred cell type labeling; and $H(T)=-{\sum}_s\frac{b_s}{n}\mathit{\log}\left(\frac{b_s}{n}\right)$ is the entropy function for true cell type labeling. We used the *compare* function in the *igraph* R package (v1.0.0) to compute both ARI and NMI criteria. For rare cell type identification, we used the *F*-measure that is commonly used for quantifying rare cell type identification performance [54, 55]. The *F*-measure is the harmonic mean of the clustering’s precision and recall, and is formulated as:

$$ F-\mathrm{measure}=2\frac{P\ast R}{P+R}. $$

where *P* represents the precision for identifying the rare cluster, with $P=\frac{\mathrm{True}\ \mathrm{Positive}}{\mathrm{True}\ \mathrm{Positive}+\mathrm{False}\ \mathrm{Positive}}$, while *R* represents the recall for identifying the rare cluster, with $R=\frac{\mathrm{True}\ \mathrm{Positive}}{\mathrm{True}\ \mathrm{Positive}+\mathrm{False}\ \mathrm{Negative}}$. For each data set, we repeated the above procedure five times and report the averaged results to avoid the influence of the stochasticity embedded in some dimensionality reduction methods and/or the clustering algorithm.

While it is straightforward to apply different dimensionality reduction methods to most scRNA-seq data sets, we found that many dimensionality reduction methods are not computationally scalable and cannot be directly applied for clustering analysis in two large-scale scRNA-seq data sets we examined in the present study. For these non-scalable dimensionality reduction methods, we made use of a recently developed sub-sampling procedure described in *dropClust* to scale them to large data [59]. In particular, we first applied dropClust to the original large-scale data to infer rare cell populations. We then created a small data by combining all cells in the rare cell populations along with a subset set of cells in the remaining cell populations. The subset of cells in the non-rare populations is obtained through sub-sampling using the structure preserving sampling procedure (details in [59]). Afterwards, we applied different dimensionality reduction methods to the small data and performed clustering analysis there. The cells in the small data are then directly assigned with their clustering label after clustering analysis. For each cell that is not in the small data, we computed the Pearson correlation between the cell and each of the cluster centers inferred in the small data. We assigned the cell to the cluster with the closest cluster center in the small data as the cluster assignment.

For trajectory inference, after dimensionality reduction with these dimensionality reduction methods, we used *Slingshot* [56] (R package, v1.2.0) and Monocle3 [28] (R package, v0.1.2). The *Slingshot* software is the recommended lineage inference method based on a recent comparative study [14]. Monocle3 is one of the most recent lineage inference methods. Slingshot takes two input data: the low-dimensional components extracted from dimensionality reduction methods and a vector of cluster labels predicted by clustering algorithms. Monocle3 also takes two input data: the low-dimensional components extracted by dimensionality reduction methods and starting state which is to the beginning of the lineage. For the cluster labels, we used either *k*-means, hierarchical clustering algorithm, or Louvain method on the extracted low-dimensional components to obtain cluster labels. For the starting state, we supplied with the true beginning state of the lineage in the data. After obtaining the two types of input through the *slingshot* function, we used the *getLineages* function to fit a minimum spanning tree (MST) to identify lineage. The final output from *Slingshot* is an object of class *SlingshotDataSet* that contains the inferred lineage information. We follow the original *Slingshot* paper [56] to evaluate the accuracy of the inferred lineage using the Kendall rank correlation coefficient. To do so, for each data, we first ranked genes based on their position on the true lineage. We ordered all *m* genes based on this rank order and denoted the corresponding rank in ascending order for these genes as {*x*_1_, ⋯, *x*_*m*_}, where *x*_*i*_ ≤ *x*_*i* + 1_. Note that the true lineage is linear without any bifurcation or multifurcation patterns, while the inferred lineage may contain multiple ending points in addition to the single starting point. Therefore, for each inferred lineage, we examined one trajectory at a time, where each trajectory consists of the starting point and one of the ending points. In each trajectory, we ranked genes in order based on their position in the trajectory. We denote the corresponding rank order in the inferred trajectory for all *m* genes as {*y*_1_, ⋯, *y*_*m*_}, where we set *y*_*l*_ as missing if *l*th gene is not included in the inferred trajectory. For each pair of non-missing genes, we labeled the gene pair (*i*, *j*) as a concordant pair if their relative rank in the inferred lineage are consistent with their relative rank in the true lineage; that is, either (*x*_*i*_ ≥ *x*_*j*_ & *y*_*i*_ ≥ *y*_*j*_) or (*x*_*i*_ < *x*_*j*_ & *y*_*i*_ < *y*_*j*_). Otherwise, we labeled the gene pair (*i*, *j*) as discordant. We denoted *C* as the number of concordant pairs, *D* as the number of discordant pairs, and *U* as the total number of non-missing genes. The Kendell correlation coefficient is then computed as

$$ \tau =\frac{C-D}{U\left(U-1\right)/2}. $$

Afterwards, we obtained the maximum absolute *τ* over all these trajectories as the final Kendall correlation score to evaluate the similarity between the inferred lineage and the true lineage. For each data set, we repeated the above procedure five times and report the averaged results to avoid the influence of the stochasticity embedded in some dimensionality reduction methods and/or the lineage inference algorithm. For the large-scale data application to Cao et al., we also applied the sub-sampling approach dropClust to scale different dimensionality reduction methods for lineage inference.

We investigated the stability and robustness of different dimensionality reduction methods in both cell clustering and lineage inference applications through data splitting. Here, we focused on two representative scRNA-seq data sets, the *Kumar* data set for cell clustering, and the *Hayashi* data set for lineage inference. For each data, we randomly split the data into two subsets with an equal number of cells in each cell type in the two subsets. We repeated the split procedure 10 times to capture the potential stochasticity during the data split. In each split replicate, we applied different dimensionality reduction methods to analyze each subset separately. We used *k*-means clustering algorithm to infer the clustering labels in each subset. We used NMI to measure cell clustering accuracy and used Kendall correlation to measure lineage inference accuracy.

Finally, to summarize the performance of the evaluated dimensionality reduction methods across the range of criteria in Fig. 5, we consider either “good,” “intermediate,” or “poor” to categorize the dimensionality reduction methods for each criterion. For UMI and non-UMI based data in cell clustering, we evaluated the performance of different dimensionality reduction methods based on 0.5% low-dimensional components in Additional file 1: Figure S31A and S31B: average NMI ≥ 0.73 (good); 0.64 ≤ average NMI < 0.73 (intermediate); average NMI < 0.64 (poor). For Trajectory Inference, we evaluated the performance of different dimensionality reduction methods based on 2 low-dimensional components in Additional file 1: Figure S39A: average Kendall ≥ 0.41 (good); 0.35 ≤ average Kendall < 0.41 (intermediate); average Kendall < 0.35 (poor). For Rare Cell Detection, we evaluated the performance of different dimensionality reduction methods based on 0.5% low-dimensional components in Additional file 1: Figure S35A: F-measure ≥ 0.74 (good); 0.69 ≤ F-measure < 0.74 (intermediate); F-measure < 0.69 (poor). For Neighborhood Preserving, we evaluated the performance of different dimensionality reduction methods based on 0.5% low-dimensional components in Additional file 1: Figure S7A: average Jaccard index ≥ 0.15 (good); 0.12 ≤ average Jaccard index < 0.15 (intermediate); average Jaccard index < 0.12 (poor). For Scalability, we evaluated the performance of different dimensionality reduction methods when sample size is 10,000 in Fig. 4b: computation time ≤ 0.25 h (good); 0.25 h ≤ computation time < 10 (intermediate); computation time ≥ 10 h (poor). For Consistency, we evaluated the performance of different dimensionality reduction methods based on the absolute mean value of the difference of average NMI between two splits from Additional file 1: Figure S36 and S54: difference of average NMI ≤ 0.005 (good); 0.005 ≤ difference of average NMI < 0.01 (intermediate); difference of average NMI ≥ 0.01 (poor). For Success Rate, since both scScope and LTSA do not work for most trajectory inference data sets, we set as poor; NMF, ICA, tSNE, and GLMPCA do not work for some of data sets, we set as intermediate; the rest of dimensionality reduction methods are all good.

## Supplementary information

**Additional file 1.** Supplementary figures and tables.

**Additional file 2.** Review history.

## Data Availability

All source code and data sets used in our experiments have been deposited at www.xzlab.org/reproduce.html or https://github.com/xzhoulab/DRComparison [85].
