# Supplementary material for: Accuracy, robustness and scalability of dimensionality reduction methods for single-cell RNA-seq analysis
Source: Genome Biol. 2019 Dec 10;20:269. doi: 10.1186/s13059-019-1898-6 (PMC6902413; doi:10.1186/s13059-019-1898-6)
Supplement: Supplementary file 2 — Additional file 2. Review history. [file 13059_2019_1898_MOESM2_ESM.docx]

Review History

**First round of review**

**Reviewer 1**

**Are you able to assess all statistics in the manuscript, including the appropriateness of statistical tests used?**

Yes, and I have assessed the statistics in my report.

**Comments to author:**

This manuscript compared 11 dimension reduction methods on 28 scRNA-seq datasets. The performances were compared for two major downstream tasks: clustering and lineage inference. The major issues:

1) The authors should consider some measurements purely for dimension reduction (such as explained variations and neighborhood preserving). The performances of downstream applications cannot fully reflect the performances of dimension reductions. Different DR methods may be suitable for different clustering and lineage inference methods. In the manuscript, only k-means/hierarchical clustering/slingshot were used. Commonly used methods, such as SNN-Cliq and Monocle were not compared.

2) The DR methods and the testing datasets should be better grouped. Different methods may only suit for some specific datasets or tasks. Especially, scientists have more concern on rare cell sub-populations rather than the general performance comparison.

3) The commonly used embedding methods such as t-SNE & UMAP (also methods based on local sensitive hash) were not investigated.

4) The practical guideline is too simple. DR methods may couple with sub-sampling to improve their applications to large-scale datasets.

5) It is suggested that the authors select one or two datasets to do in-depth analysis: which method can give more biological meaningful results.

Overall, this work lacks of in-depth analysis. Dimension reduction methods cannot be simply compared by downstream clustering/lineage inference performances.

**Reviewer 2**

**Are you able to assess all statistics in the manuscript, including the appropriateness of statistical tests used?**

Yes, and I have assessed the statistics in my report.

**Comments to author:**

In this manuscript, Xiang Zhou and colleagues provide a comparative evaluation of a variety of commonly used dimensionality reduction (DR) methods for scRNAseq analysis. The performance of each DR method is qualified by normalized mutual information (NMI) and adjusted rand index (ARI) for cell clustering analysis and Kendall correlation coefficient for trajectory inference. The computational scalability of those methods is also evaluated as well. This work will give scRNA-seq algorithm developer, and also general audience, a practical guideline for choosing DR methods. The author concluded that, for clustering analysis, PCA, ICA, FA and ZINBWaVE are recommended for small data and PCA, ICA, FA are also recommended for large data. For lineage inference analysis, FA, NMF and ZINB-WaVE are recommended for small data and FA is also recommended for large data. It provides a valuable resource for the community. However, there are many critical concerns need to be addressed before it is accepted to be published:

Major concerns:
1.     Since the single-cell analysis field is rapidly evolved, the datasets choose in this study is not representative enough for the future analysis. Firstly, in the majority of the dataset chosen, the number of cells is less than or equal to 10,000. However, nowadays the number of single cell RNA-seq study is usually more than 10,000. In my opinion, "large sample size" described in this paper is not large enough. Notably, [1] have profiled the transcriptomes of around 2 million cells in a single study. Secondly, UMI-based technology, for example, 10X genomics, and non UMI-based one, for example, Smart-Seq2, should be discussed separately. 10X genomics-like platform usually generate large scale, zero inflated dataset, which is with almost no amplification bias. At the meanwhile, the datasets generated from Smart-Seq2-like technology are usually small scale, but have high capture rate. The unwanted variation from the two types of dataset is quite different. To my knowledge, correct me if I am wrong, the largest published dataset generated from Smart-Seq2 is [2] (about 12,346 T cells). Thus, I think the author should at least include datasets from [1] and [2] in their comparison to be more informative.
2.     All the manifold learning method for dimensionality reduction are not examined in this study. For example, Multidimensional Scaling (MDS), Isomaps, Locally linear embedding (LLE), Laplacian eigenmap (LE), Local tangent space alignment (LSTA), Hessian Eigenmapping (HLLE). The authors should include some of those methods, or at least discuss why those methods are not covered.

Minor concerns:
1. Ideally, the computation time of different algorithms should be indicated by time complexity. If that is no doable, for fair comparison, different algorithms should be implemented in the same programming language. For example, R or Python. If not, the authors should tell readers the potential effect of different programming language used for each algorithm. In addition, for Figure 4A and 4B, the line for PCA (light blue) and scScope (purple) is missing or invisible.

2. A better understanding of DR methods is needed. For instance, ICA is a linear DR method. If you use a nonlinear version of ICA, you should stress it by using words "nonlinear ICA" instead of just "ICA".

3. All formulas and mathematical symbols in this paper need to be carefully examined. For instance, there are some obvious errors in formulas and mathematical symbols in ARI and NMI criteria. Formulas and mathematical symbols should be concise and accurate.

4. Abbreviation errors occurred in this paper. For example, the abbreviation of "deep learning" is "DL" instead of "LD" in figure 5.

Reference:
1. Cao J, Spielmann M, Qiu X, Huang X, Ibrahim DM, Hill AJ, Zhang F, Mundlos S, Christiansen L, Steemers FJ, Trapnell C, Shendure J: The single-cell transcriptional landscape of mammalian organogenesis. Nature 2019.
2. Guo X, Zhang Y, Zheng L, Zheng C, Song J, Zhang Q, Kang B, Liu Z, Jin L, Xing R, Gao R, Zhang L, Dong M, Hu X, Ren X, Kirchhoff D, Roider HG, Yan T, Zhang Z: Global characterization of T cells in non-small-cell lung cancer by single-cell sequencing. Nature Medicine 2018, 24:978-985.

**Reviewer 3**

**Are you able to assess all statistics in the manuscript, including the appropriateness of statistical tests used?**

Yes, and I have assessed the statistics in my report.

**Comments to author:**

The authors compare different dimensionality reduction methods (DR) by measuring their impact on typical scRNA-seq data analyses, like clustering and lineage reconstruction.
The manuscript is well written and easy to follow. The comparisons are provided on real and simulated datasets. The following are my concerns:

While the idea to divide datasets into two main groups accordingly to the final comparison task (clustering or trajectory analysis) is fine, it is not clear why authors decided for these datasets. From a benchmarking point of view, the sample identification is crucial, therefore authors should explain which features are in these data, making them suitable for the comparison. Can authors discuss if cellular data structures in these samples fit the benchmarking purpose?

Authors performed the clustering by applying two methods, k-means (km) and hierarchical clustering (hc). As shown, the clustering performance was overall lower in the case of the hc, although consistent in these cases. Could the clustering approach makes the advantage for some method with respect to others? Authors should address this point and explain better why they chose these clustering approaches.

Authors compared 11 DR methods, PCA, ICA, FA and other methods specifically designed for single cell analysis. However, I see very important the inclusion of tSNE and UMAP as they represent the two most used DR approaches by the single cell community.

**Authors Response**

**Point-by-point responses to the reviewers’ comments:**

Comment

*Response*

Reviewer: 1#

General Comment:
This manuscript compared 11 dimension reduction methods on 28 scRNA-seq datasets. The performances were compared for two major downstream tasks: clustering and lineage inference. The major issues:

*Response: Thank you very much for the constructive comments and valuable suggestions, which have significantly improved the quality of the paper. Responses to your specific comments are listed below:*

Specific comments:
1. The authors should consider some measurements purely for dimension reduction (such as explained variations and neighborhood preserving). The performances of downstream applications cannot fully reflect the performances of dimension reductions. Different DR methods may be suitable for different clustering and lineage inference methods. In the manuscript, only k-means/hierarchical clustering/slingshot were used. Commonly used methods, such as SNN-Cliq and Monocle were not compared.

*Response: Thank you for the comments. Following your first suggestion, we have added neighborhood preserving as a measurement purely for measuring the effectiveness of dimensionality reduction in terms of preserving the original features in the gene expression matrix. We did not use the other measurement suggested by the reviewer, the explained variations, for two reasons: (1) One of these DR methods, PCA, is specifically designed to maximize the explained variation. Therefore, PCA will always yield the maximized variation among all linear DR methods. (2) It is not straightforward to define explained variations for count based models (e.g. ZINB-WaVE, pCMF, Poisson NMF, DCA, and a few others). In addition, we have also explored the use of another measurement, reconstruction error, for measuring the effectiveness of dimensionality reduction in terms of preserving the original features. However, we found that reconstruction error (1) is not straightforward to define for count-based models (e.g. ZINB-WaVE , pCMF, Poisson NMF, DCA, and other DR methods) and (2) is an unstable measurement for several non-linear DR methods (e.g. LTSA, LLE and a few others). Therefore, we only added neighborhood preserving as the pure DR measurement. The new results based on the pure DR measurement are shown in Supplementary Figures S2-S14, with details explained in the Materials and Methods section (lines 96-116 on pages 25-26) and Results section (lines 22-69 on pages 5-6). We also note, however, that this newly added measurement is purely for DR purpose and may not be relevant for single cell analytic tasks that are the main focus of the present study: a DR method that preserves the original gene expression matrix effectively may not be effective in extracting useful biological information from the expression matrix that is essential for key downstream single cell applications. Preserving the original gene expression matrix is rarely the sole purpose of DR methods for single cell applications: indeed, the original gene expression matrix (which is the best-preserved matrix of itself) is rarely, if ever, used directly in any downstream single cell applications including clustering and lineage inference, even though it is computationally easy to do so. We have also added this caveat of using pure DR measurement in the Results section (lines 70-82 on pages 6-7).

Following your second suggestion, we have added SNN-Cliq and Monocle3 into comparison. The comparison results based on Monocle3 are added to a new section in Results (lines 361-379 on pages 14-15) and are largely consistent with the comparison results based on Slingshot obtained previously. The comparison results based on SNN-Cliq revealed that SNN-Cliq is overly sensitivity to the number of low-dimensional components and may incorrectly assign all cells to a single cluster. Therefore, while we report the results for SNN-Cliq in the updated Results (lines 160-179 on page 9), we have to rely on the other two commonly used and stable clustering algorithms for scRNAseq analysis (k-means and hierarchical clustering) in the main comparison. Finally, we note that, while the comparative results are largely consistent among different clustering methods and among different leakage inference methods, we fully agree with the reviewer and acknowledge that it is in principle possible some DR methods may work well with certain clustering approaches and/or with certain lineage inference approaches. We have added this important information to the Discussion section (lines 8-20 on page 21).*

2. The DR methods and the testing datasets should be better grouped. Different methods may only suit for some specific datasets or tasks. Especially, scientists have more concern on rare cell sub-populations rather than the general performance comparison.

*Response: Thank you for the comments. Following your first suggestion, we have grouped different DR methods and testing datasets into different categories. Specifically, for DR methods, we grouped 18 DR methods based on different criteria including the nature of the algorithm (e.g. linear vs non-linear; generic vs single cell specific), whether modeling counts, whether modeling zero inflation, and efficiency, as well as implementation language. These detailed classifications for the DR methods are provided in the updated Table 1. In addition, we also grouped these 18 DR methods based on their performance in clustering performance in UMI and non-UMI data, performance on trajectory inference, performance on rare cell type detection, performance on neighborhood preserving, scalability, computing speed, consistency, and success rate. These DR method performance categorizations are displayed in the updated Figure 5. For testing datasets, we also grouped the 30 testing datasets based on different criteria including sequencing techniques, UMI vs non-UMI, sample size, and studied species etc. The detailed classifications for the testing datasets are provided in the updated Supplementary Tables 1-2.

Following your second suggestion, we have added new comparisons for rare cell population detection. These results are shown in the new Supplementary Figures S30-S31, with details explained in the Results section (lines 254-283 on pages 11-12). Performance of different DR methods for rare cell population detection is also summarized in the updated recommendation figure Figure 5.*

3. The commonly used embedding methods such as t-SNE & UMAP (also methods based on local sensitive hash) were not investigated.

*Response: Thank you. Following your suggestion, we have added these two methods, t-SNE and UMAP, into all our comparisons. We have now increased the total number of compared DR methods from 11 to 18 in the updated manuscript. Figures and Results are all updated accordingly.*

4. The practical guideline is too simple. DR methods may couple with sub-sampling to improve their applications to large-scale datasets.

*Response: Thank you. Following your suggestion, we obtained two newly published large-scale scRNAseq data sets: Guo et al. data for clustering analysis and Cao et al. data for lineage inference. These two data sets contain more than ten thousand single cells (data details provided in the updated Supplementary Tables 1-2). Because many DR methods are not computationally scalable to these data sets, we also followed your suggestion and applied a recently published sub-sampling approach (dropClust) to make all DR methods scalable to large data. What we found was that, the sub-sampling approach, while being computationally efficient, substantially reduces the performance of many DR methods. Therefore, while we report these new results in the updated Results section, we also recommend users to first explore the effectiveness of the sub-sampling approach in their own data before blindly adapting the approach. The new results are provided in the Supplementary Figures S48-S50, with details provided in a newly added section in Results (lines 442-493 on pages 17-18). We also summarized these results in the updated recommendation Figure 5.*

5. It is suggested that the authors select one or two datasets to do in-depth analysis: which method can give more biological meaningful results.

*Response: Good point. Following your suggestion, we have performed in-depth analysis and visualization on two scRNAseq data sets, Kumar et al. (for cell clustering) and Zhang et al. (for trajectory inference). The new results are provided in Supplementary Figures 28 and 37, with details provided in two separate places in the Results section (lines 227-253 on page 11; lines 380-393 on page 15). These new results can complement well our main results on methods comparison, which is the focus of the Special Issue of Benchmarking Studies.*

------------------
Reviewer: 2#
General Comments:
In this manuscript, Xiang Zhou and colleagues provide a comparative evaluation of a variety of commonly used dimensionality reduction (DR) methods for scRNAseq analysis. The performance of each DR method is qualified by normalized mutual information (NMI) and adjusted rand index (ARI) for cell clustering analysis and Kendall correlation coefficient for trajectory inference. The computational scalability of those methods is also evaluated as well. This work will give scRNA-seq algorithm developer, and also general audience, a practical guideline for choosing DR methods. The author concluded that, for clustering analysis, PCA, ICA, FA and ZINBWaVE are recommended for small data and PCA, ICA, FA are also recommended for large data. For lineage inference analysis, FA, NMF and ZINB-WaVE are recommended for small data and FA is also recommended for large data. It provides a valuable resource for the community. However, there are many critical concerns need to be addressed before it is accepted to be published:

*Response: Thank you for the positive comments and constructive suggestions, which have significantly improved the quality of the manuscript. Responses to your specific comments are listed below:*

Specific Comments:
1.Since the single-cell analysis field is rapidly evolved, the datasets choose in this study is not representative enough for the future analysis. Firstly, in the majority of the dataset chosen, the number of cells is less than or equal to 10,000. However, nowadays the number of single cell RNA-seq study is usually more than 10,000. In my opinion, "large sample size" described in this paper is not large enough. Notably, [1] have profiled the transcriptomes of around 2 million cells in a single study. Secondly, UMI-based technology, for example, 10X genomics, and non UMI-based one, for example, Smart-Seq2, should be discussed separately. 10X genomics-like platform usually generate large scale, zero inflated dataset, which is with almost no amplification bias. At the meanwhile, the datasets generated from Smart-Seq2-like technology are usually small scale, but have high capture rate. The unwanted variation from the two types of dataset is quite different. To my knowledge, correct me if I am wrong, the largest published dataset generated from Smart-Seq2 is [2] (about 12,346 T cells). Thus, I think the author should at least include datasets from [1] and [2] in their comparison to be more informative. .

*Response: Thank you, and we fully agree. Following your first suggestion, we have included both these two large-scale data sets [1] and [2] into the comparison. Briefly, we found that, when directly applied to analyze the data, UMAP is computationally efficient and consistently outperforms the remaining DR methods across a range of low-dimensional components by a large margin. We found that many DR methods are not scalable to these two large-scale data sets. Because many DR methods are not computationally scalable, we applied a recently published sub-sampling approach (dropClust) to make these DR methods scalable. However, we also found that, the sub-sampling approach, while being computationally efficient, substantially reduces the performance of multiple DR methods. Therefore, while we report these new results of large-scale data analysis in the updated results, we also recommend the users to first explore the effectiveness of the sub-sampling approach in their own data before blindly adapting the approach. The new results are provided in Supplementary Figures S48-S50, with details provided in a newly added section in Results (lines 442-493 on pages 17-18). We have updated the summary figure Figure 5 to reflect these new results.

Following your second suggestion, we have now added a new section in the Results to examine the DR method performance in UMI-based technology versus non-UMI based technology. These new results are shown in Supplementary Figures S8 and S27, with details explained in the newly added section in Results (lines 52-58 on page 6; lines 199-253 on pages 10-11).*

2.All the manifold learning method for dimensionality reduction are not examined in this study. For example, Multidimensional Scaling (MDS), Isomaps, Locally linear embedding (LLE), Laplacian eigenmap (LE), Local tangent space alignment (LSTA), Hessian Eigenmapping (HLLE). The authors should include some of those methods, or at least discuss why those methods are not covered.

*Response: Thank you. Following your suggestion, we have added all these manifold learning methods for our comparison. The number of compared DR methods increased from 11 to 18 in the updated manuscript. Results are updated accordingly.*

3.Ideally, the computation time of different algorithms should be indicated by time complexity. If that is no doable, for fair comparison, different algorithms should be implemented in the same programming language. For example, R or Python. If not, the authors should tell readers the potential effect of different programming language used for each algorithm. In addition, for Figure 4A and 4B, the line for PCA (light blue) and scScope (purple) is missing or invisible.

*Response: Thank you. Following your comment, we tried our best to compare these methods in the same programming language, R, for as many DR methods as possible. In the end, we were able to compare 14 out of the 18 DR methods in R. However, 4 remaining methods (ZIFA, pCMF, scScope and UMAP) are all implemented in Python without any R implementation. We are hesitated to implement these methods in R as (1) these methods are relatively complicated and (2) our R implementation may not be as efficient as the original Python implementation. Therefore, we list the programming language for all DR methods in the updated Table 1. In addition, we also emphasize in the updated Results section that the computing time difference between these two groups may partially reflect the difference in programing language (R vs Python; lines 496-499 on page 18). In addition, we have also tried our best to obtain the time complexity for these DR methods. However, we found it challenging to do so for all DR methods, as it requires knowing the implementational details of different DR methods, some of which can be quite complex. Therefore, instead of providing this information in the manuscript, we instead list what we are able to obtain in the following table.

For your comment on the missing line of PCA in Figure 4A and the missing line of scScope in Figure 4B, these lines appear to be missing because these two methods have very similar computing time as compared to a few other DR methods. Specifically, PCA is similar to ICA in Figure 4A; and scScope is similar to several other efficient methods in Figure 4B. We have updated the Figure 4 legend to explain this important issue.

Attached table:
No. Methods Complexity ImplementationLanguage
1 PCA O(max(n^2 p+p^3,np^2+n^3)) R
2 ICA O(dnlogn+d^2 n) R
3 FA -- R
4 NMF -- R
5 Poisson NMF -- R
6 Diffusion Map O(n^2 p) R
7 ZIFA O(npB^2) Python
8 ZINB-WaVE -- R
9 GLMPCA -- R
10 pCMF -- R
11 scScope -- Python
12 DCA -- Python
13 tSNE O(n^2 p+n^2 h) R
14 MDS O(n^2 (p+d)) R
15 LLE O(plog(k)nlog(n))+O(〖nk〗^3 p)+O(n^2 d) R
16 LTSA O(plog(k)nlog(n))+O(〖nk〗^3 p)+O(k^2 d)+O(n^2 d) R
17 Isomap O(n^2 (p+log⁡(n)) R
18 UMAP O(〖dn〗^1.14) Python
Notations in runtime complexity: n: number of observations; p: number of features in the original data; k: the selected number of nearest neighbors; h: the number of iterations. d: output dimensions; B: block size.*


4.A better understanding of DR methods is needed. For instance, ICA is a linear DR method. If you use a nonlinear version of ICA, you should stress it by using words "nonlinear ICA" instead of just "ICA".

*Response: Thank you for pointing out this error. ICA is indeed a linear DR method. We have correct this error in the revised version (e.g. in Table 1).*

5.All formulas and mathematical symbols in this paper need to be carefully examined. For instance, there are some obvious errors in formulas and mathematical symbols in ARI and NMI criteria. Formulas and mathematical symbols should be concise and accurate.

*Response: We apologize for these errors, which we have corrected in the updated manuscript. We realized that we copied these formulas from a previous publication and that these formulas there were incorrect. (We used R functions to compute these criteria so the incorrect formulas in the previous text did not affect results.) We have also carefully re-examined all formulas and symbols throughout the text.*

6.Abbreviation errors occurred in this paper. For example, the abbreviation of "deep learning" is "DL" instead of "LD" in figure 5.

*Response: We apologize for this mistake, which has been corrected in the updated version. We have also carefully re-examined abbreviations throughout the text.*

------------------
Reviewer: 3#
General Comments:
The authors compare different dimensionality reduction methods (DR) by measuring their impact on typical scRNA-seq data analyses, like clustering and lineage reconstruction. The manuscript is well written and easy to follow. The comparisons are provided on real and simulated datasets. The following are my concerns:

*Response: Thank you for the positive comments and constructive suggestions, which have significantly improved the quality of the manuscript. Detailed responses are listed below.*

Specific Comments:
1. While the idea to divide datasets into two main groups accordingly to the final comparison task (clustering or trajectory analysis) is fine, it is not clear why authors decided for these datasets. From a benchmarking point of view, the sample identification is crucial, therefore authors should explain which features are in these data, making them suitable for the comparison. Can authors discuss if cellular data structures in these samples fit the benchmarking purpose?

*Response: We apologize for not explaining our criteria in selecting these datasets well in the previous manuscript. We previously followed the criteria listed in ref [12,40] to select the datasets for cell clustering analysis. In particular, these data sets need to contain known cell clusters that come from, for example, mixing different cell types through FACS sorting. In addition, we previously followed the criteria listed in ref [14] to select datasets for trajectory inference. In particular, these data sets need to contain known linear lineage information. We have obtained publicly available single cell data that are published in the past 5 years and that also fit these criteria into our study. Following your suggestion, we have now provided these detailed selection criteria in the updated Materials and Methods section (lines 16-19 and lines 25-28 on page 23).*

2. As shown, the clustering performance was overall lower in the case of the hc, although consistent in these cases. Could the clustering approach makes the advantage for some method with respect to others? Authors should address this point and explain better why they chose these clustering approaches.

*Response: Thank you for your comments. We previously selected both k-means clustering and hierarchical clustering into our analysis because of two reasons: both of these methods are key ingredient of commonly applied single cell clustering methods; both of these methods are computationally efficient and stable, thus enabling comparable across a diverse range of dimensionality reduction methods. Specifically, the k-means clustering is a key ingredient of commonly applied scRNAseq clustering methods such as SC3 and Waterfall. While the hierarchical clustering is a key ingredient of commonly applied scRNAseq clustering methods such as CIDR and CHETAH. Following your comments, we have now added another commonly used single cell clustering algorithm, SNN-Cliq, into comparison in the revised manuscript. Unfortunately, the comparison results based on SNN-Cliq revealed that SNN-Cliq is overly sensitivity to the number of low-dimensional components and may incorrectly assign all cells to the same cluster. Therefore, while we report the results for SNN-Cliq in the updated Results (lines 160-179 on page 9), we have to rely on the other two commonly used and stable clustering algorithms, k-means and hierarchical clustering, for the main comparison.

We also note that, while the comparative results are largely consistent among different clustering methods, we fully agree with the reviewer and acknowledge that it is possible some DR methods may work well with certain clustering approaches and/or with certain lineage inference approaches. We have added this important information to the Discussion section (lines 8-20 on page 21).*

3. Authors compared 11 DR methods, PCA, ICA, FA and other methods specifically designed for single cell analysis. However, I see very important the inclusion of tSNE and UMAP as they represent the two most used DR approaches by the single cell community.

*Response: Thank you and we fully agree. We have added both tSNE and UMAP, along with a five other DR methods into our comparison. The number of examined DR methods now increased from 11 to 18. All Figures and Results are updated accordingly.*

**Second round of review**

**Reviewer 1**

The manuscript made extensive revisions and addressed most of the proposed questions.

**Reviewer 2**

General comments:
The authors compared various commonly used dimensionality reduction (DR) methods on 30 scRNAseq datasets that cover a range of sequencing techniques and sample sizes. Performances of these DR methods are evaluated by two major downstream analyses: clustering and trajectory inference, and a purely evaluation for DR: neighborhood preserving. It’s very helpful for researchers to choose relevant DR methods in their study. The manuscript has been significantly improved and addressed most of the reviewer’s question. However, there are some details need to be further improved before it is accepted to be published.

Specific comments:
1. Concerns about tSNE: from line 24 to line 69, it is declared that 18 DR methods are applied and the results are summarized in Figuers S2-S14. But readers can only find 17 DR methods in these figures without tSNE. Besides, in the line 155, it is claimed that tSNE can only yield two low-dimesional components. The statement is incorrect. Thus, please check figures and sentences about tSNE carefully.

2. A suggestion of SNN-Cliq: authors only apply SNN-Cliq on one dataset due to the heavy computation burden of SNN-Cliq, that makes the results are not so convincing. I suggest authors use Louvain algorithm instead of SNN-Cliq and apply it on different datasets.

3. A suggestion of visualization: visualization is also an important part in scRNAseq analysis and authors compared the performances of DR methods in visualization from line 227 to 253. I suggest authors organize visualization discussions as an independent chapter.

4. Concerns about formulas: formulas and mathematical symbols from line 144 to 156 of chapter “METHODS AND MATERIALS” need to be carefully examined. For instance, line 145, the mathematical symbols P=(p_1,p_1,…,p_n) should beP=(p_1,p_2,…,p_n); line 146, the mathematical symbols T=(t_1,t_1,…,t_n) should be T=(t_1,t_2,…,t_n); line 148, the formula n_l=∑_l▒〖I(p_i=l)〗 should be n_l=∑_i▒〖I(p_i=l)〗; line 149, the formula n_s=∑_s▒〖I(t_i=s)〗 should be n_s=∑_i▒〖I(t_i=s)〗. Also in line 151, n_ls need to be modified because it’s Irrelevant about indictors l and s after the sum but relevant about i, besides n_ls constantly equal to 1.

5. Some spelling error: For example, the word “read” should be “real” in line 13 of the “RESULTS”.

**Authors Response**

**Point-by-point responses to the reviewers’ comments:**

Reviewer: 2#

General Comment:
The authors compared various commonly used dimensionality reduction (DR) methods on 30 scRNAseq datasets that cover a range of sequencing techniques and sample sizes. Performances of these DR methods are evaluated by two major downstream analyses: clustering and trajectory inference, and a purely evaluation for DR: neighborhood preserving. It’s very helpful for researchers to choose relevant DR methods in their study. The manuscript has been significantly improved and addressed most of the reviewer’s question. However, there are some details need to be further improved before it is accepted to be published.

*Thank you very much for the constructive comments and valuable suggestions, which have significantly improved the quality of the paper. Responses to your specific comments are listed below:*

Specific comments:
1. Concerns about tSNE: from line 24 to line 69, it is declared that 18 DR methods are applied and the results are summarized in Figuers S2-S14. But readers can only find 17 DR methods in these figures without tSNE. Besides, in the line 155, it is claimed that tSNE can only yield two low-dimesional components. The statement is incorrect. Thus, please check figures and sentences about tSNE carefully.

*Response: Thank you for the comments and we apologize for the mistake on tSNE. Indeed, tSNE can be used to yield either two low-dimensional components or three low-dimensional components in practice. In addition, at least in principal, tSNE can also be used to extract more than two or three low-dimensional components. Unfortunately, we haven’t been able to find a software package that can stably and reliably extract more than three low-dimensional components. In particular, we tried many existing t-SNE fitting software used in standard single cell software packages and these t-SNE fitting software can only yield either a maximum of two low-dimensional components (e.g. FIt-SNE) or a maximum of three low-dimensional components (e.g. Rtsne and Rdimtools). These aforementioned t-SNE fitting software are the backbones of many single cell analysis and visualization packages. For example. Seurat offers three different options for t-SNE fitting: Rtsne which is the Barnes-Hut implementation; Flt-SNE which is a fast FFT-accelerated interpolation implementation; and the standard t-SNE that is not recommended for large data. As another example, Monocle uses Rtsne. In the present study, we also used the most recent method Flt-SNE (Linderman et al., Nature Methods 2019; which only extracts two low-dimensional components) for all our analysis done here. Due to this practical limitation of existing t-SNE fitting software especially the fast Flt-SNE package used here, we had to perform t-SNE analysis using only two low-dimensional components. In addition, we previously did not include the t-SNE results in the figures (hence 17 DR methods in these figures) and only explained the t-SNE results in the main text, because t-SNE results can only be displayed in one column (for two-dimensional components) while other methods can be displayed in four columns (for four different choices of the number of low-dimensional components).

Following your comment, we recognized that our previous approach of dealing with t-SNE results was not ideal. Therefore, following your suggestion, we have added the t-SNE results back to all figures and supplementary figures. In addition, we have added a brief explanation in line of above on why we only displayed the two-dimensional components results for t-SNE in all figures in both the Results section (lines 20-21 on page 5) and in the Materials and Methods section (lines 93-96 on page 25).*

2. A suggestion of SNN-Cliq: authors only apply SNN-Cliq on one dataset due to the heavy computation burden of SNN-Cliq, that makes the results are not so convincing. I suggest authors use Louvain algorithm instead of SNN-Cliq and apply it on different datasets.

*Response: Thank you. Following your suggestion, we have replaced SNN-Cliq with Louvain algorithm to all data sets. These results are shown in the new Supplementary Figures S20-S22, S25, 26C, S29, S30C, S38, S39C, S45, S46C, S49, and S50C, with details explained in the Results section (lines 164-187 on pages 9-10; lines 293-300 on page 13; lines 364-380 on page 15).*

3. A suggestion of visualization: visualization is also an important part in scRNAseq analysis and authors compared the performances of DR methods in visualization from line 227 to 253. I suggest authors organize visualization discussions as an independent chapter.

*Response: Good point. Following your suggestion, we have added two subtitles (line 236 on page 11; line 400 on page 16) so that the visualization results are separated into independent chapters/subsections.*

4. Concerns about formulas: formulas and mathematical symbols from line 144 to 156 of chapter “METHODS AND MATERIALS” need to be carefully examined. For instance, line 145, the mathematical symbols P=(p_1,p_1,…,p_n) should beP=(p_1,p_2,…,p_n); line 146, the mathematical symbols T=(t_1,t_1,…,t_n) should be T=(t_1,t_2,…,t_n); line 148, the formula n_l=∑_l▒〖I(p_i=l)〗 should be n_l=∑_i▒〖I(p_i=l)〗; line 149, the formula n_s=∑_s▒〖I(t_i=s)〗 should be n_s=∑_i▒〖I(t_i=s)〗. Also in line 151, n_ls need to be modified because it’s Irrelevant about indictors l and s after the sum but relevant about i, besides n_ls constantly equal to 1.
(note that the equations in the reviewer’s comments may not display well when pasted into the journal submission link.)

*Response: We apologize for these errors, which we have corrected in the updated manuscript. We have also carefully re-examined all formulas and symbols throughout the text.*

5. Some spelling error: For example, the word “read” should be “real” in line 13 of the “RESULTS”.

*Response: We apologize for this mistake, which has been corrected in the updated version. We have also carefully read throughout the text and eliminated other potential typos.*

**Third round of review**

**Reviewer 2**

The manuscript made extensive revisions and addressed most of the proposed questions.
